# 454 Pyrosequencing to Describe Microbial Eukaryotic Community Composition, Diversity and Relative Abundance: A Test for Marine Haptophytes

**DOI:** 10.1371/journal.pone.0074371

**Published:** 2013-09-12

**Authors:** Elianne Egge, Lucie Bittner, Tom Andersen, Stéphane Audic, Colomban de Vargas, Bente Edvardsen

**Affiliations:** 1 University of Oslo, Department of Biosciences, Marine Biology, Oslo, Norway; 2 CNRS UMR7144 & UPMC, Station Biologique de Roscoff, Roscoff, France; 3 University of Oslo, Department of Biosciences, Integrative Biology, Oslo, Norway; 4 University of Kaiserslautern, Department of Ecology, Kaiserslautern, Germany; University of Connecticut, United States of America

## Abstract

Next generation sequencing of ribosomal DNA is increasingly used to assess the diversity and structure of microbial communities. Here we test the ability of 454 pyrosequencing to detect the number of species present, and assess the relative abundance in terms of cell numbers and biomass of protists in the phylum Haptophyta. We used a mock community consisting of equal number of cells of 11 haptophyte species and compared targeting DNA and RNA/cDNA, and two different V4 SSU rDNA haptophyte-biased primer pairs. Further, we tested four different bioinformatic filtering methods to reduce errors in the resulting sequence dataset. With sequencing depth of 11000–20000 reads and targeting cDNA with Haptophyta specific primers Hap454 we detected all 11 species. A rarefaction analysis of expected number of species recovered as a function of sampling depth suggested that minimum 1400 reads were required here to recover all species in the mock community. Relative read abundance did not correlate to relative cell numbers. Although the species represented with the largest biomass was also proportionally most abundant among the reads, there was generally a weak correlation between proportional read abundance and proportional biomass of the different species, both with DNA and cDNA as template. The 454 sequencing generated considerable spurious diversity, and more with cDNA than DNA as template. With initial filtering based only on match with barcode and primer we observed 100-fold more operational taxonomic units (OTUs) at 99% similarity than the number of species present in the mock community. Filtering based on quality scores, or denoising with PyroNoise resulted in ten times more OTU_99%_ than the number of species. Denoising with AmpliconNoise reduced the number of OTU_99%_ to match the number of species present in the mock community. Based on our analyses, we propose a strategy to more accurately depict haptophyte diversity using 454 pyrosequencing.

## Introduction

Haptophytes are a major component in marine pico- and nanoplankton communities, occurring in all seas as important primary producers [1]. Blooms of haptophytes may have a major impact on the global carbon balance and on climate forcing [2], and may cause fish-kills [3], [4]. Knowledge on the haptophyte diversity, distribution and dynamics at the species level is, however, limited because they are small and fragile, and species identification usually requires electron microscopy or molecular biological methods, except for a few easily recognisable bloom formers such as *Emiliania huxleyi* and *Phaeocystis* spp. During monitoring surveys by light microscopy haptophytes are usually identified only to genus level (e.g. [5]). Because physiological and ecological functionalities, e.g. growth preferences and tolerances, growth rate, nutrition, swimming behaviour, toxicity, and life cycle differ among species [3], [6], there is a strong need for more efficient and accurate methods to identify and quantify haptophytes at the species level in order to better understand their ecological and economical roles.

Molecular methods are increasingly used for identification of protists in samples from natural environments, and have the potential to detect small, fragile and rare species that may perish during sampling and fixation for morphological identification [7]. Sequencing ribosomal RNA genes (rDNA) has become a standard for assessing diversity of microorganisms in samples from different environments and target groups [8], [9]. With the advent of next generation sequencing (NGS) technologies, such as 454 pyrosequencing, producing thousands of sequence reads per sample, came the opportunity to go deeper into microbial communities than what is feasible with clone libraries and Sanger sequencing. NGS methods may thus reveal the rare organisms, the so-called “rare microbial biosphere” [10]–[12]. Recent studies using Sanger sequencing of clone libraries as well as NGS suggest that also within the Haptophyta there exists a high diversity of unknown and uncultured species in marine plankton communities [13]–[18].

To assess the ecological importance of an organism, and determine the community structure (composition, abundance and distribution of the components), the abundance of the organisms is of interest in addition to merely the presence or absence [19]. The proportion of reads from NGS has been assumed to correlate with the proportion of marker copies of a given organism relative to co-occurring taxa (discussed by e.g. Amend et al. [20]). More recently, standardising read abundance data with counts of an internal standard by qPCR has been suggested as a method to estimate absolute sequence numbers in natural samples [21], [22]. However, to estimate cell numbers or biomass of a taxon, neither the ‘proportional abundance’ nor ‘internal standard’ approaches can overcome biases arising from variable copy number of ribosomal genes among taxa [20], [23], taxon-specific DNA extraction [24] or PCR amplification [25], [26].

Isolating RNA and sequencing cDNA reverse-transcribed from rRNA, instead of rDNA is a strategy which circumvents the bias due to differences in rDNA copy number among taxa [27]. The rRNA content per cell also varies between and within taxa (e.g. [28]), but to a lesser degree than rDNA copy number, and has been found to positively correlate with growth rate and cell volume in phytoplankton [28]–[30]. Environmental sequencing of rRNA/cDNA may thus more reflect the activity and production rate of the organisms, and give different results with respect to relative abundance of taxa and species diversity compared to rDNA [24], [27].

Initial environmental pyrosequencing studies suggested a richness of operational taxonomic units (OTUs) considerably higher than previously observed with clone libraries [10], [12], [19]. However, sequencing errors produced by NGS techniques are now known to generate spurious phylotypes that inflate estimates of OTU richness [31], [32], and the large number of reads produced means that the absolute number of noisy reads may be considerable [33]. Several bioinformatic methods have been developed to detect and reduce errors. These methods include removing reads with errors in known parts of the read such as the primers and multiplex identifiers (MIDs), end-trimming of reads based on the quality scores provided by the sequencer [34], improved base-calling algorithms (denoising of flowgrams) [35], algorithms to correct single-base PCR errors [33], [35], and improved OTU clustering methods [32], [36]. Generally, a combination of these approaches is recommended to reduce spurious diversity and infer the number of OTUs in a sample [34].

In Haptophyta the full-length nuclear SSU rDNA region has been shown to be a good phylogenetic marker down to the species level [37], [38]. However, as the maximum read length of the current 454 pyrosequencing technology is about 400–500 bp, hypervariable regions of the SSU rDNA such as the V4 is often used as a marker for the taxonomic classification. Some closely related morphological species differ only in a few of the c. 1800 bp in the full-length SSU (e.g. within the genus *Prymnesium*), and certain species such as *Emiliania huxleyi* and *Gephyrocapsa oceanica* have identical SSU rDNA sequences. Clustering V4 SSU rDNA sequences into groups <99% similarity may therefore mask some of the real haptophyte diversity. Both filtering and clustering methods imply a trade-off between keeping taxonomic resolution and limiting spurious diversity. Evaluating how sequence filtering methods affect the resulting dataset used to infer diversity, community composition and relative abundance is therefore important.

Our overall aim is to investigate the ability of 454 pyrosequencing to depict the haptophyte diversity in a controlled sample (mock community) and assess proportional abundances at the species level. We conducted a preliminary study on this mock community, where we constructed partial LSU rDNA clone libraries from DNA and RNA. Results from this study indicated that the proportional species distribution in a sequence library may be different from the proportional distribution in terms of both cell number and biomass, and that it may vary depending on the primary nucleic acid source used (DNA or RNA). The methods used and the results obtained in the preliminary study are described in Supporting Information File S1. We explored the mock community further using 454 pyrosequencing, addressing the following questions: *i*) Do proportion of rDNA and rRNA pyrosequencing reads correlate to proportion of cell number or biomass of the haptophyte species present in the mock community? *ii*) Is there a difference between targeting the rRNA gene (rDNA) and the rRNA (cDNA from rRNA) with respect to detection of species and estimation of relative abundance of the different species? *iii*) How can we process 454 pyrosequencing reads to keep as much of the taxonomic information as possible while minimising spurious phylotypes, in order to better infer species composition and diversity?

To answer these questions, we constructed an artificial community of haptophytes consisting of equal numbers of cells from cultures of 11 haptophyte species representing all major orders, including three closely related species within the genus *Prymnesium.* One million cells from each culture were pooled, and total DNA and RNA extracted, PCR-amplified and 454-sequenced. To test possible amplification bias caused by the affinity of the primers we designed two different haptophyte-specific primer pairs targeting the V4 region of the SSU rDNA. Differences in DNA content and rDNA copy number per cell could affect the relative read abundance among the species in the mock community. We tested the importance of this effect by including a sample in which DNA was extracted separately from each of the 11 species and pooled in equal amounts (by weight) prior to pyrosequencing.

Further, we tested four different strategies to filter the 454 pyrosequences from errors, based on both properties of the reads, and flowgram denoising methods (PyroNoise [35] and AmpliconNoise [33]). This study proposes a strategy to test, evaluate and apply 454 pyrosequencing for diversity studies of protists in general and haptophytes specifically. We also highlight problems associated to the assessment of diversity and taxa abundances using 454 pyrosequencing.

## Materials and Methods

### Preliminary Study with Clone Libraries

The protocol and results from the preliminary study can be found in File S1. Briefly, the mock community of haptophytes was constructed and DNA and RNA extracted as described below. In addition to the extraction method used in the main study we tested mechanical lysis of the cells by bead-beating. PCR-products from cDNA transcribed from RNA extracted with bead-beater appeared weaker and more fragmented than PCR-products from cDNA/RNA extracted without (Figure S1). Extraction with bead-beater did not increase the number of species recovered in the corresponding clone libraries (Figure S2, Table S1). The 454 pyrosequencing samples were therefore produced from DNA and RNA extracted without bead-beater.

### Mock Community of Haptophytes

The mock community consisted of 11 strains of different species representing six orders of Haptophyta (Table 1), obtained from the University of Oslo Culture Collection of Algae. The cultures were grown in volumes of 25 ml in borosilicate tubes with algal medium IMR ½ [39] supplemented with 10 nM selenium, or ES-medium [40] at 30 or 34 PSU. Growth conditions were identical to those of the stock cultures of each species (16 or 19°C, under white fluorescent light at a photon flux rate of 50–100 µmol m^−2^ s^−1^ and a 12∶12 h light:dark cycle). The cultures were harvested in the exponential growth phase, 11 days after inoculation. Cell concentrations were determined using a Fuchs-Rosenthal counting chamber under a light microscope (Zeiss, Axio Scope A1, Germany), counting one chamber per culture (as a rule >400 Lugol’s fixed cells). A volume (0.1–10 mL) corresponding to 1^.^10^6^ cells from each culture was transferred to a 50 ml centrifuge tube, and the cells were concentrated by centrifugation (Sorvall, RT7), 4000 rpm at 4°C for 30 min. The supernatant was removed until c. 2.5 ml liquid was left at the bottom of the tube. The pelleted cells and remaining liquid were frozen and stored at −80°C until further processing.

**Table 1:** Summary of Haptophyta strains included in the mock community.

Order	Species	Strain	Algal medium	Cell concentration (cells mL^−1^)	Avg. cellvolume (µm^3^)	Avg. celldiameter (µm)	Accession no SSUrDNA	Accession no LSU rDNA
Prymnesiales	*Chrysochromulina throndsenii*	UIO 048	IMR½, 30	1325000	147.8	6.6	AJ246279	AM779759.1
Pavlovales	*Diacronema ennorea*	UIO 021	IMR½, 34	875000	118.8	6.1	JF714242	JF718754.1
Isochrysidales	*Emiliania huxleyi*	UIO 061	ES, 30	1285000	37.7	4.2	AF184167	EU502880.1
Prymnesiales	*Haptolina fragaria*	UIO 029	IMR½, 30	97500	135.3	6.4	AM491013	**AM850683**
Prymnesiales	*Imantonia rotunda*	UIO 138	IMR½, 34	10933333	22.4	3.5	AJ246267	**AM779757**
Isochrysidales	*Isochrysis galbana*	UIO 102	IMR½, 34	3060000	54.0	4.7	AJ246266	EU729474.1
Phaeocystales	*Phaeocystis globosa*	UIO 062	ES, 30	1285000	22.6	3.5	AF182111	nd
Coccolithales	*Pleurochrysis pseudoroscoffensis*	UIO 094	IMR½, 34	75500	1285.4	13.5	AM490973	EU502877.1
Prymnesiales	*Prymnesium kappa*	UIO 033	IMR½, 30	900000	104.3	5.8	AJ246271	**AM850685**
Prymnesiales	*Prymnesium parvum*	UIO 054	IMR½, 30	775000	308.1	8.4	AJ246269	AM850698.1
Prymnesiales	*Prymnesium polylepis*	UIO 036	IMR½, 30	22188	415.7	9.3	**AJ004866**	AM850688

The strains are maintained at the University of Oslo Culture Collection of Algae. Algal medium were IMR ½ at salinity 30 or 34, or ES at salinity 30 as indicated. Cell volumes were determined by CASY measurements, and cell diameter estimated assuming a spherical cell shape. Accession numbers are of the strains used (bold) or of a different strain of the same species. nd = no data.

Average cell size in each of the cultures was measured in an electronic particle counter (CASY, Schärfe System, Germany). As electrolyte we used sterile filtered seawater with salinity as in the culture analysed. Five measurements of 200 µl were done for each species, and the size distribution and average cell volume (µm^3^) was estimated. Cell concentrations and cell sizes are listed in Table 1.

### DNA/RNA Extraction and cDNA Synthesis

The pellet was thawed on ice, and the cells were resuspended until homogenized. DNA and RNA were extracted with the RNA NucleoSpin II kit (Macherey-Nagel, Düren, Germany), with the NucleoSpin RNA/DNA Buffer set and addition of β-mercaptoethanol in the lysing step. To each of five parallel eppendorf tubes containing 350 µl of the sample, 350 µl lysis buffer and 3.5 µl β-mercaptoethanol were added, the tubes were vortexed for 30 s, and RNA and DNA was extracted according to the protocol from the manufacturer. From each of the extraction columns DNA was eluted with 100 µl DNA elution buffer and RNA with 60 µl RNase free H_2_O. The parallel DNA and RNA eluates were pooled, and the concentrations measured with a NanoDrop spectrophotometer (Wilmington, DE, USA). Before reverse transcription the RNA was treated with DNase (TURBO DNase kit, Ambion, Austin, TX, USA), according to the protocol from the manufacturer.

cDNA was synthesised from RNA with the AccuScript High-Fidelity 1st Strand cDNA Synthesis Kit (Agilent, Santa Clara, CA, USA) with random primers (octamers), according to the protocol from the manufacturer. For the synthesis reaction approximately 100 ng of RNA was added to a mix containing 2.0 µl AccuScript RT Buffer 10×, 3 µl random primers (0.1 µg µl^−1^), 0.8 µl dNTP (25 mM each dNTP) and RNase-free water to a total volume of 16.5 µl. The mix was incubated at 65°C for 5 min before annealing at room temperature for 5 min. Subsequently 2 µl 100 mM DTT (dithiothreitol, reducing agent), 1 µl AccuScript Reverse Transcriptase and 0.5 µl RNase Block ribonuclease inhibitor were added. The reaction was incubated at 25°C for 10 min before cDNA synthesis took place at 42°C for 60 min in a temperature-controlled heating block (QBT2, Grant Instruments, UK). The synthesis reaction was terminated by incubation at 70°C for 15 min.

### Preparation of ‘DNApool’

DNA was also extracted from each of the 11 species separately, following the protocol described above, quantified with NanoDrop and pooled in equal amounts (8.5 ng) of DNA from each species. In the following this sample will be referred to as ‘DNApool’.

### V4 SSU Ribosomal DNA and cDNA Amplification and 454 Pyrosequencing

Two haptophyte-specific primer pairs targeting the V4 region of the SSU rDNA were designed to give nucleotide fragments of about 400 bp suitable for 454 pyrosequencing (Table 2). The primers were designed or modified by eye from an alignment including all available unique haptophyte SSU rDNA sequences from cultures downloaded from EMBL (all publicly available as of 01.01.2012 but omitting duplicates), aligned in MAFFT v.6 with the Q-ins-i strategy [41]. Representative sequences from other major marine planktonic groups were included to ensure sufficient mismatches with non-target groups. The forward primer in primer pair Prym454, PRYM03+3, was designed by modification of the probe PRYM03 [42], and is specific to the class Prymnesiophyceae, but has mismatches to members of the class Pavlovophyceae. The reverse primer in primer pair Prym454, HaptoR1, was designed in this study for specific amplification of the entire division Haptophyta. Primer pair Hap454 consists of forward primer 528Flong, which was modified from primer 528F [43], and reverse primer PRYM01+7, which was modified from the probe PRYM01 [44] to be specific for division Haptophyta. PRYM01+7 matched 100% with sequences from all haptophyte species in the alignment. The overlap of the regions spanned by the two primer pairs was about 250 bp, PRYM03+3 being positioned 150 bp upstream of 528Flong. A distance analysis based on alignments of the SSU rDNA sequences of the strains present in the mock community, truncated at the primer pair positions, shows that both primer pairs theoretically can resolve closely related species differing in 4 bp or more (Table S2A, S2B).

**Table 2:** PCR primers for 454 pyrosequencing, targeting the V4 region of SSU rDNA.

Primer	Strand	Primerpair	Target	Sequence (5′-3′)	Position rel. to sequence AJ004866 (*Prymnesium* *polylepis* SSU rDNA)	Reference
528Flong	F	Hap454	Haptophyta	GCGGTAATTCCAGCTCCAA	572–590	This study, modified from [43]
PRYM01+7	R	Hap454	Haptophyta	GATCAGTGAAAACATCCCTGG	949–969	This study, modified from [44]
PRYM03+3	F	Prym454	Prymnesiophyceae	GTAAATTGCCCGAATCCTG	432–450	This study, modified from [42]
HaptoR1	R	Prym454	Prymnesiophyceae	CGAAACCAACAAAATAGCAC	824–843	This study.

Fusion primers for pyrosequencing were designed according to the protocol by Roche by adding adaptors (Adaptor A (5′-3′): CCATCTCATCCCTGCGTGTCTCCGAC, adaptor B (5′-3′): CCTATCCCCTGTGTGCCTTGGCAGTC), key (TCAG), and MIDs to the SSU V4 primers, which were RP-cartridge purified after synthesis (Eurogentec, Seraing, Belgium). The fusion primers were tested *in vitro* by running PCR under conditions as described below, on DNA extracted from each of the strains separately, resulting in successful amplification of all species as verified by gel-electrophoresis (result not shown). PCR for 454 pyrosequencing was performed with the fusion primers directly, on an Eppendorf thermocycler in 25-µl reactions containing 5 × Phusion GC buffer, 0.4 µM of each primer, 0.2 mM dNTP, DMSO 3%, 0.5 U Phusion polymerase (Finnzymes, Vantaa, Finland) and c. 4 ng DNA as template or 2 µl of the cDNA synthesis reaction described above. The PCR-program was as follows: initial denaturation step at 98°C for 30 s, then 30 cycles of 98°C for 10 s, 55°C for 30 s, 72°C for 30 s, and final extension at 72°C for 10 min. For each primer pair and template combination (two primer pairs and template DNA or cDNA), PCR was run in four separate reactions. The PCR-products were pooled and run on a 1% agarose gel (SeaKem, Philadelphia, PA, USA), cut out of the gel and purified using the Wizard SV gel and PCR-clean up system kit (Promega, Madison, WI, USA), according to the protocol from the manufacturer. The purified amplicons were quantified with NanoDrop and pooled in equal concentrations prior to emulsion PCR and pyrosequencing.

Emulsion PCR and pyrosequencing of the amplicons were performed at the Norwegian Sequencing Centre at the Department of Biology, University of Oslo (www.sequencing.uio.no), using GS-FLX Titanium technology. The amplicons were prepared for sequencing with Lib-L chemistry and were sequenced unidirectionally, from the forward primer, on ¼ of a 454 life sciences FLX Titanium sequencing plate (454 Life Sciences, Branford, CT, USA), in multiplex with another study. 454 GS FLX flowgrams (.sff files) were deposited in the European Nucleotide Archive with accession number ERP002556 (http://www.ebi.ac.uk/ena/data/view/ERP002556).

### Filtering of 454 Pyrosequencing Reads

We tested four different sequence-filtering procedures. The effects of these filtering methods were evaluated with respect to 1) the proportion of reads left after filtering 2) the number of OTUs (operational taxonomic units) at a given clustering level 3) the proportion of reads assigned to each reference sequence (termed proportional species distribution) 4) the proportion of unassigned reads (i.e. assigned to taxonomic levels higher than species) 5) the percentage mismatch between a reference sequence and the reads that are assigned to it (error rate). Unless stated otherwise, the procedures were performed in mothur v. 1.23 (www.mothur.org) [45].

#### Procedure 1: Initial Filtering (IF)

The reads were subjected to an initial filtering based on match with the MID and forward primer and sorted into groups by the trim.seqs-command, to remove reads with features known to be associated with sequencing errors. We used the following settings: No mismatches with the MID or forward primer (bdiffs = 0, pdiffs = 0), as recommended by [33], [46], which is also the default setting in the first step of AmpliconNoise, no ambiguous base calls (Ns) (maxambig = 0), maximum length of homopolymer; 8 (maxhomop = 8), and minimum read length 365 bp (minlength = 365). By truncating the reference alignment at various positions and calculating the pairwise distances between the truncated sequences, we found that a read length of 365 bp was sufficient to keep the original taxonomic resolution. In Haptophyta, the targeted SSU V4 region contains a 6 bp homopolymer, and maxhomop = 8 was chosen to allow for some flexibility around this length. To ensure that the filtered reads covered the same region in the alignment space, they were aligned with ‘align.seqs’, with the k-mer search method against a template alignment, (the same which was used for primer design), k-mer size 8, and the Needleman alignment method. Reads that did not align in the V4 region were removed with the screen.seqs-command. Putative chimeras were identified by the ‘chimera.perseus’ subroutine in mothur, an implementation of the Perseus algorithm [33]. The reads flagged as chimeric were removed from the datasets.

#### Procedure 2: Quality Score filtering (QS)

The reads that had passed procedure 1, up to the chimera checking, were subjected to trimming based on the quality scores of each base call provided from the sequencer. Average quality scores over a moving window of 50 bp were calculated by the trim.seqs-command, with the settings qwindowaverage = 30 and qwindowsize = 50. Once the average quality score over a region dropped below 30, the read was trimmed to the end of the last window with an average score at or above this limit. After trimming, reads that were shorter than 365 bp were removed from the dataset, and the remaining sequences were aligned as described in procedure 1. Putative chimeras were identified and removed from the datasets using the same method as in procedure 1.

#### Procedure 3: PyroNoise (PN)

The flowgrams were filtered as in procedure no. 1 with ‘trim.flows’ to remove flowgrams that had mismatches with the MID and the forward primer, homopolymers longer than 8 bp, and that consisted of less than 360 or more than 720 flows. Denoising of the original flowgrams was then done by the algorithm PyroNoise [35] as implemented in ‘shhh.flows’ in mothur. PyroNoise clusters flowgrams at a given threshold by complete linkage hierarchical clustering based on flowgram distance, and infers the most probable sequence that generated the flowgrams in the cluster by an expectation-maximization algorithm [33]. We used the default setting for the flowgram preclustering step, which is to cluster flowgrams with a distance smaller than 0.01. After denoising, sequences that were shorter than 365 bp were removed, and the remaining sequences were aligned as described in procedure 1. Putative chimeras were identified and removed from the datasets using the same method as in procedure 1.

#### Procedure no. 4. AmpliconNoise in Qiime (AN)

Processing of the original sff files was performed with AmpliconNoise as implemented in Qiime v. 1.4.0. AmpliconNoise is a development of the PyroNoise algorithm that is capable of removing 454 sequencing errors and PCR single base errors separately [33]. We used default settings, i.e. no mismatch with MID and forward primer, cluster flowgrams with a distance smaller than 0.01, cluster size 60 in the flowgram preclustering step, and cut-off for the initial clustering in SeqNoise 0.08, cluster size 30. Subsequently, reads that were shorter than 365 bp were removed. Chimera removal by Perseus is an integral part of the Qiime implementation of AmpliconNoise. Perseus was run with default settings. The sequences produced after treatment with AmpliconNoise were inspected by eye in BioEdit v. 7.1.7 and compared to the reference sequences to check for indels and mismatches.

### Taxonomic Assignation of Filtered Pyrosequencing Reads

For each of the resulting 20 datasets (2 templates × 2 primer pairs, plus ‘DNApool’ × 4 filtering procedures), the sequences were assigned to one of the 11 reference sequences (Table 1) by ‘classify.seqs’, the mothur implementation of the bayesian k-mer search [47], with k-mer size 8 and bootstrap cutoff at 70%. Each reference sequence was given a taxonomic assignation from species to division level, in accordance with the latest revisions of the taxonomy of the Haptophyta [38], [48]. If the probability score (as described in [47]) of a read assigned to two or more reference sequences was the same, the read was assigned to the lowest taxonomic level common to these sequences.

### Read Quality and Error Rate

To assess the general quality of the reads assigned to each species after ‘Initial Filtering’, from the samples DNA-Hap454 and DNA-Prym454, the average quality score for each base call was plotted against the position in the sequence.

The error rates in the sequences remaining after the different filtering procedures were calculated by the procedure seq.error in mothur. The 11 SSU rDNA sequences from the species present in the mock community (Table 1) were used as reference sequences, and the error rate was calculated as the distance between the query sequence and the corresponding reference sequence, as described in [34].

### Clustering

To examine the effect of the different filtering procedures on the OTU richness in the samples, the filtered and processed reads were clustered at similarity levels ranging from 100% to 85%. Uncorrected pairwise distances between the aligned sequences were calculated with the dist.seqs-command, with settings calc = onegap (a string of gaps counting as a single gap). The sequences were subsequently clustered with the average-neighbour algorithm [49].

### Phylogenetic Analysis

To illustrate the effect of denoising 454 pyrosequencing reads, we constructed phylogenetic trees based on unique OTU_99%_ obtained from sample DNA-Hap454 after the four filtering procedures had been applied, together with the 11 reference sequences. The analysis was done by ‘clearcut’, a mothur implementation of the Neighbor-Joining algorithm [50].

### Statistical Analyses

The chi-square statistic was used to compare the proportional species distributions obtained with the different template+primer combinations. Pearson’s product-moment correlation coefficient (r) and Kendall’s concordance coefficient (τ) were used to test the association between the proportional read abundance and proportional biomass. To investigate the differences between the proportional species distributions obtained with the different primer-template-filtering combinations, pairwise dissimilarity matrices were calculated using the Bray-Curtis dissimilarity index [51]. Dissimilarities were visualised as ordination diagrams based on non-metric multidimensional scaling (NMDS). Analysis of variance using distance matrices, ‘adonis’ [52] was used to test for significant differences in proportional species distributions obtained after the four bioinformatic filtering procedures. All statistical analyses were performed in R version 2.15.1. [53]. NMDS and ‘adonis’ were carried out using the *vegan* package [54]. Rarefaction curves were constructed using function ‘rarefaction’ [55] which makes use of the function ‘rarefy’ in *vegan*, with 5 as the interval between subsamples. ‘rarefy’ calculates the expected number of species in a subsample of the specified size, as defined in [56]. The function ‘rarefaction’ incrementally increases the size of the subsample, and plots the expected number of species as a function of subsample size. The number of reads required to get a species richness expectation equal to the true richness was estimated by inverse interpolation of the rarefaction curve.

## Results

### Taxonomic Distribution and Estimation of Relative Abundance

**Figure 1:**
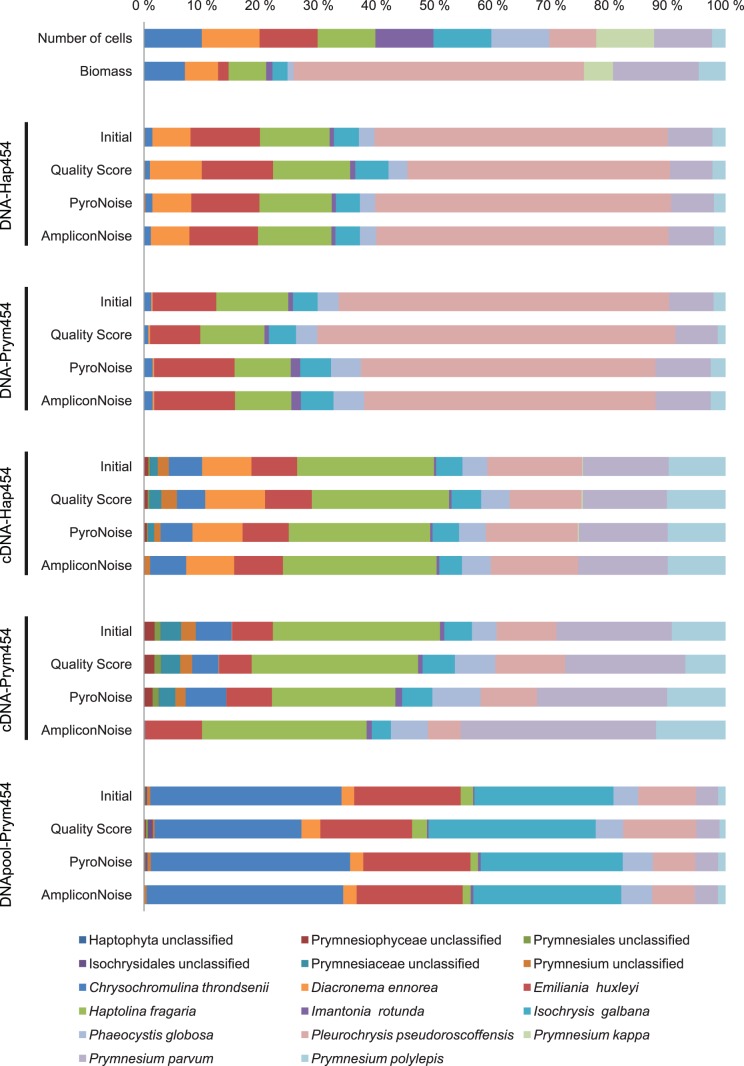
Proportional species abundance in 454 pyrosequencing reads from a mock community of haptophytes. Compared to the initial distribution of species in terms of cell number (first row) and biomass (second row) in the mock community. The different combinations of template (DNA or RNA/cDNA), primer pair, (Hap454 or Prym454), and read filtering strategy are indicated on the figure. Initial Filtering (IF): Initial filtering based on match with barcode and primer, and removal of reads with Ns and/or homopolymers >8 bp, and reads that are shorter than 365 bp. Quality Score (QS): requiring minimum average quality score 30 over a 50 bp moving window along the read, in addition to IF. PyroNoise (PN): the PyroNoise algorithm as implemented in mothur, with default settings. AmpliconNoise (AN): AmpliconNoise as implemented in Qiime v.1.4.0, with default settings. Chimera identification by Perseus was applied to all datasets. The filtering strategies are described in the methods section.

We obtained between 11 778 and 20 503 reads per sample. Overview of the samples, template+primer combinations, and the number of reads from each sample is shown in Table 3. The proportion of 454 reads in the samples assigned to each species in the mock community following the different sequence filtering procedures is shown in Figure 1. Ten out of eleven species initially added to the mock community were systematically detected in all samples and filtering conditions tested herein. Only *Prymnesium kappa,* which constituted c. 5% of the mock community biomass (Figure 1, second row, bright green), was not detected in all conditions except one; the cDNA-Hap454 sample where it constituted <0.2% of the reads. Overall, the proportional species distribution in all of the samples was significantly different from that of the initial mock community, both in terms of cell number (Figure 1, first row) and biomass (Figure 1, second row) (χ^2^- and p-values given in Table 4).

**Table 3:** Overview over the 454 pyrosequencing samples.

Sample type	Template	Primer pair	Sample name	# reads
1 million cells from each of 11 species	DNA	Hap454	DNA-Hap454	12170
“	DNA	Prym454	DNA-Prym454	17532
“	cDNA	Hap454	cDNA-Hap454	20503
“	cDNA	Prym454	cDNA-Prym454	17788
Equal amount of DNA from each species	DNApool	Prym454	DNApool	11778

**Table 4:** Comparison of proportional species distribution between the samples and the species distribution in the mock community in terms of cell number and biomass.

Compared to:	Initial distribution ofbiomass from thedifferent species	Initial distributionby cell number	DNA-Prym454	cDNA-Hap454	cDNA-Prym454
**Sample**					
DNA-Hap454	χ^2^_9_ = 7289 p**<0.001**	χ^2^_9_ = 23081, p**<0.001**	χ^2^_9_ = 954, p<**0.001**(BC-dist: 0.08)	χ^2^_9_ = 3910, p<**0.001**(BC-dist: 0.38)	
DNA-Prym454	χ^2^_8_ = 9456, p**<0.001**	χ^2^_8_ = 39010, p**<0.001**			χ^2^_9_ = 7091, p<**0.001**(BC-dist: 0.49)
cDNA-Hap454	χ^2^_9_ = 14942, p**<0.001**	χ^2^_9_ = 9381, p**<0.001**			χ^2^_9_ = 1354, p<**0.001**(BC-dist: 0.15)
cDNA-Prym454	χ^2^_8_ = 16650, p**<0.001**	χ^2^_8_ = 8703, p**<0.001**			
DNApool	–	Equal proportion of eachspecies: (χ^2^_10_ = 12903,p<**0.001**)	–	–	–

χ^2^-values and corresponding p-values from comparisons of the species distributions in the samples, treated with ‘Initial Filtering’. Significant (p<0.05) differences in bold type. The Bray-Curtis dissimilarities between the samples are given in parentheses.

#### Proportional species distribution in DNA compared to cDNA

**Figure 2:**
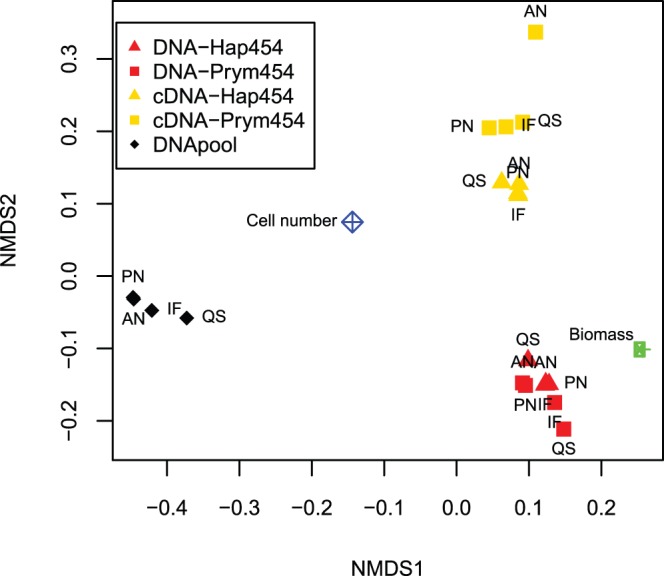
Non-metric multidimensional scaling (NMDS) of Bray-Curtis dissimilarities between the samples, after treatment with four filtering strategies. Stress value: 0.04. The green point represents the proportional distribution of species by biomass in the original mixture of cells. The blue point represents the distribution in terms of cell number. Points that cluster together represent similar proportional species distributions. Red: DNA, yellow: RNA/cDNA, Upward triangle: Primer pair Hap454, Square: Primer pair Prym454. Black diamonds represent the pool of equal amounts of DNA from each species, amplified with primer pair Prym454.

There were notable and statistically significant differences between the DNA-based and cDNA-based samples, (Figure 1, Table 4). Independently of the primer set used, the sequences from the cDNA samples were distributed more evenly amongst the species than those from the DNA samples as indicated by smaller χ^2^- values when compared to a distribution with equal number of sequences from each species (Table 4). Based on cell volume calculation, *Pleurochrysis pseudoroscoffensis* constituted the greatest proportion of biomass, and this species was also dominating in the DNA-based samples, representing c. 50% of the reads. In the cDNA-based samples *Haptolina fragaria* was dominating with c. 24–29% of the reads, whereas *P. pseudoroscoffensis* only constituted 10–16%. This main difference in proportional taxonomic distribution between the DNA- and cDNA-based samples is reflected in the ordination diagram of Bray-Curtis dissimilarities (Figure 2), where the samples cluster primarily according to the type of template.

**Figure 3:**
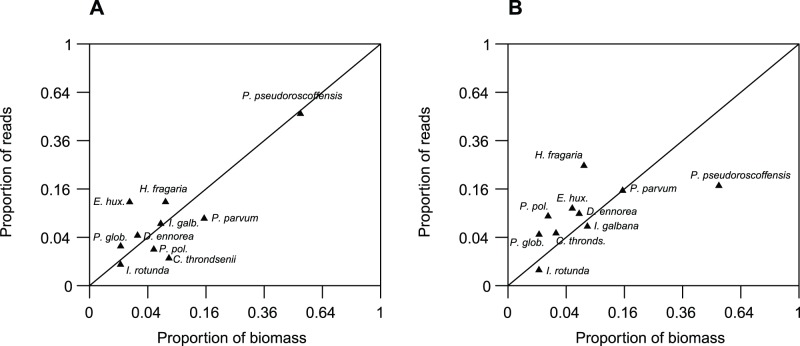
Correlation between proportional species abundance among the reads and the proportional species abundance by biomass in the mock community. (A) Based on DNA as a template in the PCR, (B) based on RNA/cDNA. The coordinates for each species correspond to the proportional abundance in the biomass (x-axis) and the proportional abundance among the reads (y-axis), after ‘Initial Filtering’-treatment of the reads. The straight line *x* = *y* shows the expected proportion of reads based on proportion of biomass. Species that lie below the line are proportionally less abundant in the reads than in the biomass, and vice versa. Note that the axes are scaled by squaring the values to better distinguish between the data points. The correlation between proportional biomass and read abundance with DNA as template was r = 0.94, p<0.001. With cDNA as template the correlation was r = 0.48, p = 0.16. When *P. pseudoroscoffensis* was omitted from the DNA-values the correlation was not significant (r = 0.24, p = 0.53). The data shown here are obtained with primer pair Hap454. We obtained a similar result with primer pair Prym454 (not shown.). Correlation between the primer pairs was r = 0.99 for DNA, r = 0.93 for cDNA.

To assess further the representation of proportional species distribution in the 454 reads compared to the biomass, we plotted proportional abundance of reads assigned to each species (*y*-axis) against proportional biomass (*x*-axis) (Figure 3). As the filtering methods in some cases affected the proportional species distribution, we used the sequence dataset obtained after ‘Initial Filtering’ for this analysis. The correlation between proportional read abundance and biomass was stronger for the DNA samples (r = 0.94, p<0.001) than for the cDNA samples (r = 0.48, p = 0.16). However, the stronger correlation for the DNA reads was mainly driven by the data point representing *P. pseudoroscoffensis.* When this point was omitted, the correlation was no longer significant (r = 0.24, p = 0.53). Further, the rank associations (Kendall’s τ) between proportional read abundance and biomass were τ = 0.42, p = 0.11, for DNA and τ = 0.64, p = 0.01 for cDNA. The stronger Pearson correlation (r) between DNA and proportional biomass is also reflected by the shorter distance from the DNA-based sample cluster to the point representing proportional biomass in the ordination diagram (Figure 2). However, as for the correlation, the ordination was also influenced by *P. pseudoroscoffensis.* When *P. pseudoroscoffensis* was omitted there was no difference in the distance between the DNA- and cDNA sample clusters and biomass (not shown).

The sequence distribution obtained from pyrosequencing the pooled DNA with equal amounts of total DNA from each species was significantly different from a distribution of equal abundance of each species (χ^2^_10_ = 12903, p<0.001, Table 4). This suggests that a bias is introduced during the PCR or pyrosequencing, which is not caused by differences in DNA concentration or rDNA copy number [26].

#### Effect of primer pair

There was a small, but significant difference between the proportional species distributions obtained with the two primer pairs (χ^2^-values in Table 4). Primer pair Hap454 matched all presently sequenced species within Haptophyta, whereas Prym454 matched all members of class Prymnesiophyceae, but had mismatches with members of Pavlovophyceae in the forward primer. As expected, Prym454 did not efficiently amplify the species representing the Pavlovophyceae, *Diacronema ennorea.* Except for this species, the two primer pairs produced similar proportional species distributions for a given template, as shown in Figures 1 and 2. The correlation in proportional species distribution between the primer pairs were r = 0.99 for DNA and r = 0.93 for cDNA, when *D. ennorea* was omitted from the data.

### Rarefaction Analysis

We constructed rarefaction curves for the ‘Initial Filtering’-treated samples to assess the number of reads required from each sample to recover all the species present (Figure S3). The curves show for each sample the expected number of species retrieved (*y-*axis) as a function of number of reads sampled (*x*-axis). A sample in which a species occurs in very low proportion will require more reads sampled to reach saturation. For cDNA-Hap454, the only sample in which *Prymnesium kappa* was present, but only with 0.16% of the reads, inverse interpolation of the rarefaction curve indicates that as much as 1400 reads were necessary to get a species richness expectation equal to the true richness (11). The other samples, in which *P. kappa* was not retrieved, reached saturation at 10 species, with between c. 700 (DNA-Hap454) and 3000 reads (cDNA-Prym454). The high number of reads needed to reach saturation in cDNA-Prym454 was due to the low proportion of *Diacronema ennorea* reads in this sample.

### Effect of Filtering Procedure

**Figure 4:**
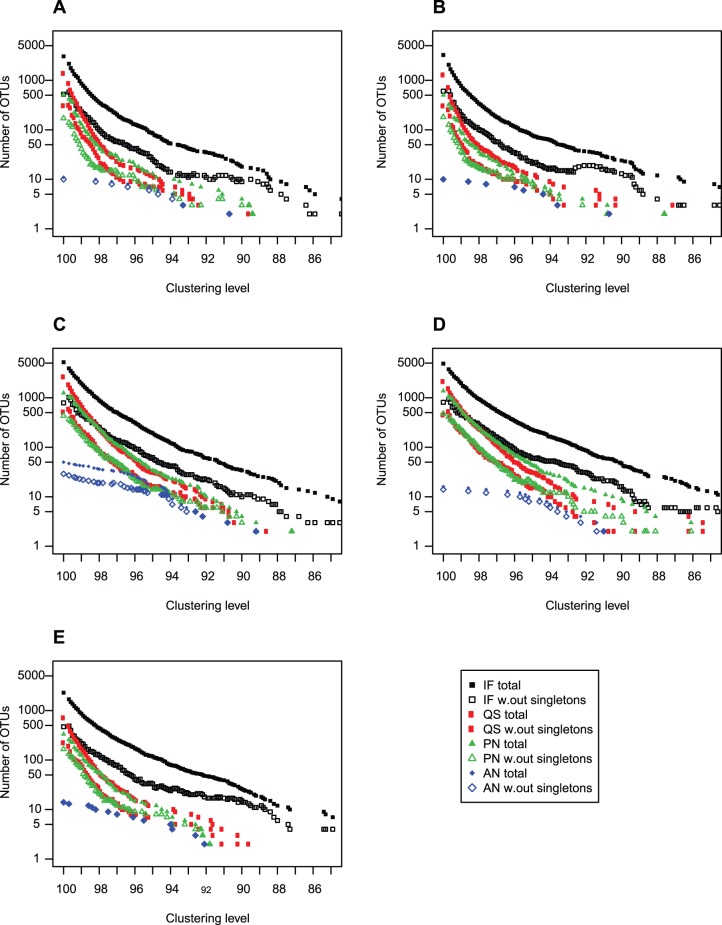
Number of OTUs as a function of clustering level and filtering procedure. The 454 pyrosequencing reads were processed with four different sequence filtering strategies, and the number of OTUs were calculated with and without singletons. (A) DNA-Hap454, (B) DNA-Prym454, (C) cDNA-Hap454, (D) cDNA-Prym454, (E) DNApool. Initial Filtering (IF): Initial filtering based on match with barcode and primer, and removal of reads with Ns and/or homopolymers >8 bp, and reads that are shorter than 365 bp. Quality Score (QS): requiring minimum average quality score 30 over a 50 bp moving window along the read, in addition to IF. PyroNoise (PN): the PyroNoise algorithm as implemented in mothur, with default settings. AmpliconNoise (AN): AmpliconNoise as implemented in Qiime v. 1.4.0, with default settings. The filtering strategies are described in the methods section.

We tested four different procedures to filter the sequence data from errors: 1. Initial Filtering (IF), 2. Quality Score filtering (QS), 3. PyroNoise (PN), and 4. AmpliconNoise (AN). The efficiency and effect of each of these were assessed by the proportion of remaining reads longer than 365 bp, the overall error rate (Table 5), the proportional species distribution in the reads after filtering (Figure 1), and the number of OTUs at clustering levels ranging from 100-85% similarity (Figure 4). After ‘Initial Filtering’, 68–88% of the sequences remained. Following Quality Score filtering 39–56% of the sequences remained, whereas fewer sequences were removed with the two denoising methods (PN and AN), keeping between 58–78% and 51–83% of the reads, respectively.

**Table 5:** Summary statistics of the 454 pyrosequencing reads and effect of bioinformatic filtering strategies.

A) Sample	# reads	# singletons (%)	# unique reads	# (%) chimeras after Initial Filtering
DNA-Hap454	12170	3986 (40%)	4684	591 (5%)
DNA-Prym454	17532	4313 (25%)	5113	306 (2%)
cDNA-Hap454	20503	8924 (44%)	10263	4263 (23%)
cDNA-Prym454	17788	8230 (46%)	9513	3093 (20%)
DNApool	11778	3516 (30%)	4250	795 (9%)
**B) Sample**	**% reads remaining after filtering. IF, QS, PN, AN**	**# OTU_99%_. IF, QS, PN, AN**	% unclassified* sequences. **IF, QS, PN, AN**	**Error rate. IF, QS, PN, AN**
DNA-Hap454	85, 50, 78, 83	858, 233, 130,11	0.20, 0.20, 0.30, 0.01	0.010, 0.008, 0.003, 0.002
DNA-Prym454	88, 56, 67, 68	769, 152, 88, 10	0.07, 0.06, 0.09, 0.00	0.006, 0.005, 0.005, 0.003
cDNA-Hap454	69, 45, 63, 71	1935, 721, 543, 42	4.29, 5.68, 2.89, 1.07	0.008, 0.006, 0.003, 0.004
cDNA-Prym454	68, 39, 58, 51	1958, 628, 557, 15	8.39, 8.29, 7.17, 0.13	0.009, 0.006, 0.005, 0.003
DNApool	79, 42, 72, 76	864, 176, 124, 12	1.10, 1.87, 1.19, 0.49	0.011, 0.009, 0.009, 0.008

(A) Number of reads, number of singletons, number of unique reads before any filtering, and proportion of chimeras detected by Perseus in the different samples. (B) Effect of filtering procedures on proportion of reads remaining after filtering, number of OTU_99%_, proportion of unclassified sequences, and error rate. ‘Unclassified’ reads are reads that could not be assigned to species level.

#### Error rates, chimeras and unclassified sequences

The overall error rates in the different combinations of sample and filtering method varied from 0.2–1.1% (Table 5). After only ‘Initial Filtering’, the error rates were between 0.6–1.1%. AmpliconNoise achieved the greatest reduction in error rate and reduced the error rates to 0.2–0.8%. The proportion of putative chimeric sequences (originating from more than one species) was higher in the reads based on cDNA (20–23%) than DNA (2–5%, 9% in DNApool, Table 5). A larger proportion of the reads (2.9–8.4%) could not be classified to species level in the cDNA-based samples compared to the DNA-based after filtering procedures 1–3, (IF, QS, PN, Table 5). These unclassified sequences could possibly be remaining chimeras not detected by Perseus. Further, the proportion of singletons in the raw data was higher in the cDNA-based samples than in the DNA-based (44 and 46% vs. 40 and 25%, Table 5). The proportion of unclassified reads was reduced to 0–1% after treatment with AmpliconNoise and Perseus, indicating that this procedure efficiently detected chimeras and reduced noise resulting in uncertain classifications.

The number of OTUs as a function of clustering level and with different filtering procedures is shown in Figure 4A–E. Based on the pairwise distances between the reference sequences (Table S2A, S2B), where 4 was the minimum bp difference over the c. 400 bp region, we considered OTUs clustered at 99% similarity as ‘species’. At 99% similarity, after only the initial filtering method where no quality score filtering or denoising was applied we obtained between 858–1958 OTU_99%_ from the mock community of 11 species (Table 5). Quality Score filtering reduced the number of OTU_99%_ by 73–80% (down to 152–233 OTUs), in the DNA-based samples, and by 63–68% (down to 628–721 OTUs), in the cDNA-based, compared to only the initial filtering procedure. PyroNoise was somewhat more efficient, reducing the number of OTU_99%_ by 85–89%, (down to 88–130), in the DNA-based and c. 72% (down to 543–557) in the cDNA based, but still not sufficiently to represent species of haptophytes. Treatment with AmpliconNoise proved efficient in reducing the number of OTU_99%_, down to 10–42. Clusters containing only one read (singletons) are known to often be a result of PCR or sequencing error, and not represent real phylotypes. Removal of singleton clusters resulted in 202–446 OTU_99%_ after IF, 54–233 after QS, 36–223 after PN and 10–22 after AN. In sample DNA-Hap454 we removed singletons, ‘doubletons’, ‘tripletons’ and so forth, to see if the number of OTU_99%_ after removing low-abundant OTU_99%_ at any point was the same as the number of species in the mock community. After all OTU_99%_ containing 10 reads or less were removed, 42 OTU_99%_ still remained in this sample after ‘Initial Filtering’. The corresponding numbers for QS, PN and AN were 19, 14 and 10 OTU_99%_, respectively. However, in sample cDNA-Prym454, *Diacronema ennorea* was only represented with 10 reads, and thus removing low-abundant (<10 reads) OTU_99%_ might remove real phylotypes. There is thus not a specific cut-off of number of reads per OTU that can be used to identify unreliable OTUs. For all samples, AmpliconNoise treatment gave the lowest number of OTUs over the relevant range of clustering levels. In both the AmpliconNoise-treated DNA samples, we obtained 10 OTU_99%,_ one corresponding to each of the species in the mock community, except *Prymnesium kappa*.

The number of indels and mismatches in the sequences produced after denoising with AmpliconNoise compared to the reference sequences are presented in Table S3. Generally, the AmpliconNoise-treated sequences match the reference sequences well, with at most one mismatch and 0–6 indels. The indels were usually associated with homopolymers >3 bp.

**Figure 5:**
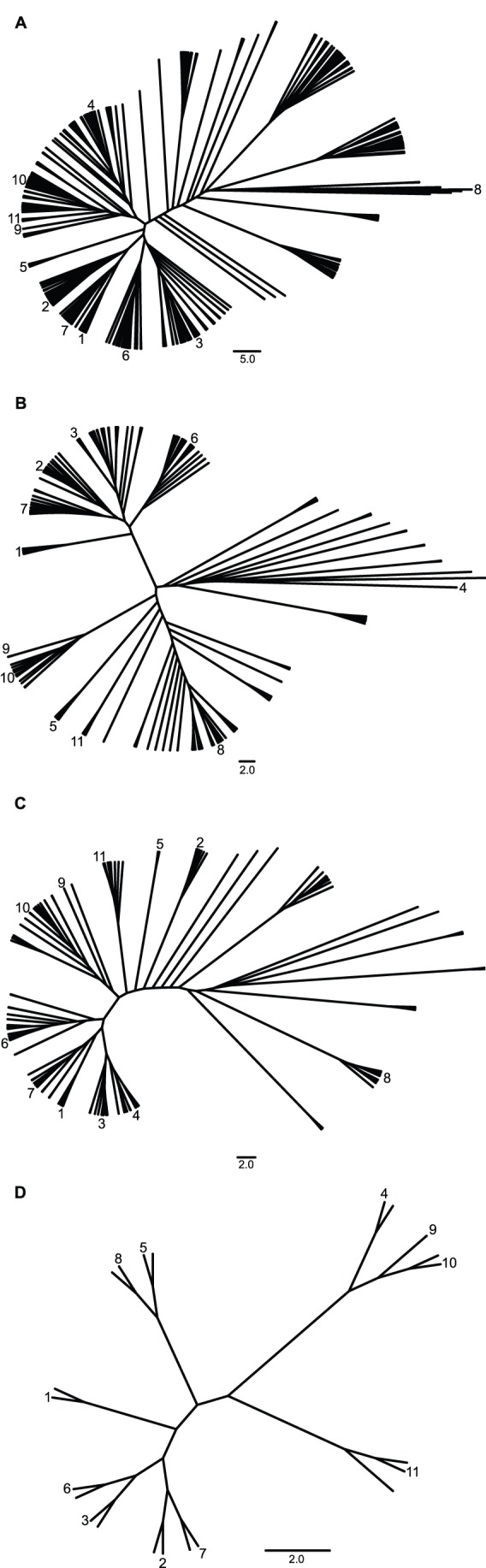
Neighbour-Joining trees of OTU_99%_ obtained after the different filtering procedures, in sample DNA-Hap454. Constructed from unique OTUs at a clustering level of 99% similarity, obtained after the filtering procedures (A) Initial Filtering, (B) Quality Score, (C) PyroNoise and (D) AmpliconNoise. The numbers next to the branches indicate the placement of the reference sequences from the strains used in this study. (1) *Chrysochromulina throndsenii* (AJ246279), (2) *Diacronema ennorea* (JF714242), (3) *Emiliania huxleyi* (AF184167), (4) *Haptolina fragaria* (AM491013), (5) *Imantonia rotunda* (AJ246267), (6) *Isochrysis galbana* (AJ246266), (7) *Phaeocystis globosa* (AF182111), (8) *Pleurochrysis pseudoroscoffensis* (AM490973), (9) *Prymnesium kappa* (AJ246271), (10) *Prymnesium parvum* (AJ246269), (11) *Prymnesium polylepis* (AJ004866).

Figure 5 shows cladograms of unrooted Neighbor-Joining trees based on unique OTU_99%_ (including singleton OTUs) from sample DNA-Hap454 and the 11 reference sequences after Initial Filtering (5A), Quality Score (5B), PyroNoise (5C) and AmpliconNoise (5D). Although QS and PN remove some of the noise resulting in the enormous diversity seen with only IF, AmpliconNoise has the most dramatic effect, reducing the number of OTU_99%_ in this sample to 11, one of which was a singleton OTU.

#### Effect of filtering method on proportional species distributions

There was not a significant effect of filtering procedure on the proportional species distributions (‘adonis’ statistic F = 0.07, 3 df, p = 0.97), however some effects could be seen for certain samples (cf. Figure 1 and 2). After treatment with AmpliconNoise, no reads in the cDNA-Hap454 sample were assigned to Prymnesium kappa, compared to 23 reads after ‘Initial Filtering’. A similar reduction was seen for Chrysochromulina throndsenii in the cDNA-Prym454 sample, to which only 6 reads (0.07%) out of 9124 were assigned after AmpliconNoise-treatment compared to 5–7% with the other filtering methods. The confidence of a base call is indicated by the quality score, and the quality score profiles were similar for all species within each primer pair (Figure S4A, S4B). Thus it is reasonable that for most samples the proportional species distribution was not particularly affected by the bioinformatic filtering method.

There were some differences among the filtering procedures in the number of reads that could not be assigned to species level after filtering (‘unclassified’ reads), especially for the cDNA samples. To assess whether unclassified reads could be chimeras not detected by Perseus, we checked the reads assigned as ‘unclassified Prymnesiophyceae’ in the cDNA samples with chimera.uchime (the mothur implementation of Uchime [57]), which allowed us to use the mock community sequences as references. Of these reads 42.5% and 25.1% (in cDNA-Hap454 and cDNA-Prym454 respectively), were flagged as chimeric. Some ‘unclassified’ reads could thus possibly be chimeras not detected by the chimera-checking algorithm. With AmpliconNoise there was a substantial reduction in the proportion of unclassified sequences, and nearly all sequences could be assigned to a species.

## Discussion

### Does Relative Read Abundance Reflect Biomass or Cell Number?

Size fractionation during sampling is often applied as a strategy to target different groups in the marine phytoplankton community. For instance the size cutoff 2 or 3 µm is commonly used to distinguish nano- and picoplankton [7]. All species in the present study fall within the nanoplankton size range (2–20 µm), and this study shows that even species that are relatively close in size and that are represented with the same number of cells in a sample may have very different proportional abundances in a sequence library. Both DNA and cDNA gave species distributions significantly different from the original distribution both in terms of biomass and cell number. The correlation between proportional read abundance and proportional biomass was stronger for the DNA-based samples than the cDNA-based; however, this was due to the large influence of *Pleurochrysis pseudoroscoffensis* in the DNA samples. RDNA copy number has been found to be correlated to genome size in eukaryotes [58], and genome size (as measured in pg) has in turn been found to correlate with cell size for species within the order Prymnesiales (Edvardsen unpublished data). Such a correlation has also been found within other taxonomic groups of phytoplankton, e.g. diatoms [59]. The predominance of sequences assigned to *Pleurochrysis pseudoroscoffensis* both in the DNA-based 454 reads and the clone libraries in the preliminary study (Figure S2) may thus be explained by the large cell size and possibly large genome and high rDNA copy number of this species. However, the rDNA copy number has not yet, to our knowledge, been reported for any haptophyte species. When *P. pseudoroscoffensis* was omitted from the analysis, there was no significant correlation between proportional biomass and proportional sequence abundance in the DNA-based samples. For a genetically diverse group such as the entire Haptophyta, correlations between cell volume, genome size and rDNA copy number may differ among groups, and other factors may also be important in determining which species will be proportionally more abundant in the reads. Further, studies have shown that there probably are differences in genome size between the two classes of haptophytes, Prymnesiophyceae and Pavlovophyceae [60]. Although the contribution of *Diacronema ennorea* to the culture mix in terms of biomass was c. 3 times larger than that of *Emiliania huxleyi,* the proportion of sequences assigned to *E. huxleyi* was 0.55 and 1.23 times the proportion of sequences assigned to *D. ennorea* in DNA-Hap454 and cDNA-Hap454, respectively. This observation may be explained by the larger genome size of the coccolith-bearing diploid stage of *E. huxleyi* compared to *D. ennorea.* Within the Pavlovophyceae, to date the genome size has been estimated only of the two strains *Diacronema gyrans* strain CCMP 607 and *Diacronema* sp. CCMP 610, and were found to be almost ten times smaller (28.7 Mbp, 20.6 Mbp, respectively) than the estimated genome sizes of *Prymnesium polylepis* (230 Mbp) [61] and *E. huxleyi* (220 Mbp) [60].

Specific growth rate is known to be inversely correlated to cell volume and carbon content within several taxonomic algal groups, i.e. smaller cells divide more frequently than large [62]. The ratio ribosomal RNA:DNA is known to increase proportionally with the growth rate [63]. Other sources of error or bias set aside, smaller species may thus be proportionally better represented in a library based on cDNA reverse transcribed from the ribosomal RNA itself than the rRNA genes (rDNA), compared to the species distribution by biomass. This may explain why *P. pseudoroscoffensis* was proportionally less abundant both in the cDNA clone libraries in the preliminary study (Figure S2), and in the 454 samples based on a cDNA template. However, for the species other than *P. pseudoroscoffensis*, there was no clear trend that the bigger species were proportionally less abundant in the cDNA-based samples compared to the DNA-based. Further, comparing proportions of species between samples can be problematic, as the relative read abundance of one species will be affected by other species in the pool [20], (i.e. proportionally more reads from one species means proportionally less of the other(s)). Thus it is not possible to say whether proportionally fewer reads of *P. pseudoroscoffensis* from cDNA reflects fewer *P. pseudoroscoffensis* ribosomes in absolute numbers, or more ribosomes from the other species, or a combination of these. This problem is analogous to the one presented in [21], Figure 1.

*Prymnesium kappa,* which constituted c. 5% of the mock community biomass, was only found in the sample cDNA-Hap454 where it constituted <0.2% of the reads. The reason for this low read number could be that reads originating from *P. kappa* were classified as *Prymnesium polylepis* or *Prymnesium parvum,* which differ from *P. kappa* in only 4, resp. 7 bp in the targeted region (Table S2A, S2B). It may also have been assigned to ‘unclassified *Prymnesium*’ or ‘unclassified *Prymnesiaceae*’. The strain of *P. kappa* was obtained by serial dilutions and may not be clonal (origination from one cell). There may be variation in the SSU rDNA within the strain, possibly both intra- and interclonal variation, indicated by many ambiguities in the ITS rDNA of this strain (UIO 033/EN3) [64]. *Prymnesium kappa* was neither detected in the DNApool sample, where all species were present with equal amounts of DNA. This indicates that low DNA content was not the main reason for the bias against *P. kappa*.

### Similar Species Distribution between the Two Primer Pairs

The similar species distribution between the two primer pairs suggests that the discrepancy between proportional biomass and proportional sequence abundance that is created during amplification and sequencing for the most part is not random, but reproduced with the two different primer pairs. This observation is in correspondence with that of Engelbrektson et al. [65], who found that within two regions of the 16S; V1–V2, and V8, the evenness (i.e. relative abundance) of dominant OTUs in a bacterial community were reproduced with different primer pairs, provided that template mismatches were accommodated for by degeneracies in the primer. High consistency in OTU composition between replicate amplifications and 454 runs has also been observed in samples from e.g. ectomycorrhizal plant root communities [66].

### Filtering Procedure has a Dramatic Effect on Spurious Diversity

The reduction in spurious diversity obtained by denoising methods is consistent with the benchmark studies [33], [34], and results from a study targeting ciliates in environmental samples [46]. The mothur implementation of PyroNoise, shhh.flows, (with setting 360–720 flows) has been shown to perform on average slightly better with respect to error rate than quality-score filtering requiring average Q30 over a 50 bp sliding window [34]. This is consistent with our finding that PyroNoise gives a lower number of spurious OTUs than the Quality Score filtering. AmpliconNoise in turn outperformed PyroNoise, which shows the importance of the SeqNoise algorithm.

Generally, AmpliconNoise did well in recreating sequences matching the reference sequences. In some instances, in particular for cDNA-Hap454, there was more than one unique sequence assigned to each of the species after AmpliconNoise treatment. In these cases the most abundant one matched the reference well, except for 1–2 indels (Table S3), whereas the less abundant, which usually only occurred in <4 copies would contain several mismatches. Thus for this dataset it seems safe to consider unique OTUs occurring in low abundances after AmpliconNoise treatment as errors, not reflecting actual diversity.

We found that 30–70% more reads were retained with the denoising methods than with the QS-filtering (Table 5B). As PN and AN are correcting sequences, and not just removing reads based on quality scores, they have the potential to salvage reads that otherwise would have been culled by QS-filtering with our settings.

### Filtering Method Affected Proportional Species Distribution

Surprisingly, reads from *C. throndsenii* were reduced from c. 700 (6.2%) to 6 (0.07%) after AmpliconNoise-treatment in the cDNA-Prym454 sample. AmpliconNoise does involve clustering of the flowgrams at 0.01 flowgram distance, however, *C. throndsenii* is 15 bp different from the most similar species in the targeted region (Table S2A, S2B), so it seems unlikely that flowgrams originating from *C. throndsenii* should have been clustered together with another species in the mock community. This effect of AmpliconNoise-treatment was not found for any of the other samples.

### Chimera Formation caused by cDNA Synthesis?

Although the number of samples is not high enough for proper statistical analysis, the two cDNA samples had considerably higher percentage of reads flagged as chimeric than the DNA samples (20–23% compared to 2–9%). cDNA-synthesis involves an additional copying and synthesis step that may produce chimeras despite that the protocol from the manufacturer is strictly followed. We used random primers (octamers), and a possible explanation for increased chimera formation is that if a random primer anneals within the targeted region, a cDNA strand is created that does not cover the full region. When a PCR primer anneals to this strand in the subsequent PCR, the first PCR cycle will generate a fragment that covers only a part of the targeted region. This fragment may function as a primer in the next cycle, but because it can be longer than a regular primer, it may anneal non-specifically, and thus a chimeric sequence will be generated in the next PCR cycle. Synthesis of an incomplete target sequence during reverse transcription will thus have the same effect as incomplete extension during PCR, which is known to cause chimera formation [67]. Modifications in the protocol, for instance cDNA synthesis with the target primers instead of random primers could possibly alleviate this problem.

## Conclusions and Recommendations

All 11 species in the mock community were retrieved by 454 pyrosequencing of the SSU rDNA V4 region, when using RNA/cDNA as template and primer pair Hap454. For a sample consisting of 11 species of haptophytes, with equal proportions in terms of cell number, rarefaction analysis suggested that 1400 reads were required to recover all of the species, with our primers and experimental setup. Relative read abundance did not correlate to relative cell numbers, and showed only a weak correlation to proportional biomass of the different species. 454 pyrosequencing data from environmental studies should therefore be interpreted with caution with respect to abundance both in terms of cell numbers and biomass of the different taxa. However, to obtain an estimate of relative abundance in terms of biomass of a haptophyte taxon, DNA as template may possibly be more appropriate than cDNA. Data on average cell size and rRNA gene copy number of species found in a sample may help to interpret the proportional read abundance in terms of proportional biomass abundance. To retrieve most of the active diversity present in a sample, cDNA as template may be the choice, although care should be taken to avoid increased chimera formation associated with cDNA synthesis.Of the filtering methods we tested, AmpliconNoise was the most efficient at removing noise, and kept the taxonomic resolution necessary to distinguish between the species in the mock community. At 99% clustering level and after removing singletons, the same number of OTUs was retrieved as the number of species in the mock community, and the AmpliconNoise-treated sequences had in most cases not more than 0–2 indels when compared to the reference sequences. Thus, to estimate the number of haptophyte species present in a sample we suggest to denoise the sequence data with AmpliconNoise or another type of denoising algorithm, and in addition cluster the sequences to an appropriate level for the gene and taxa in question. However, in one sample AmpliconNoise did affect the taxonomic distribution by reducing the abundance of one OTU, and we therefore recommend comparing the taxonomic distribution in the dataset before and after denoising.

This study suggests a strategy to more accurately depict haptophyte diversity using 454 pyrosequencing.

## Supporting Information

Figure S1 **Agarose gel loaded with PCR products for clone libraries.** From left to right: Ladder (O’GeneRuler 100 bp Plus, Fermentas), template cDNA-bb (from RNA extracted without bead-beater), cDNA+bb (from RNA extracted with bead-beater), DNA-bb (DNA extracted without bead-beater), DNA+bb (DNA extracted with bead-beater).
(PDF)

Figure S2 **Proportional species abundance in LSU rDNA/rRNA D1–D2 clone libraries.** Compared to the initial distribution of species in terms of cell number (first row) and biomass (second row) in the mock community. DNA+bb: DNA extracted with bead-beater, DNA-bb: DNA extracted without bead-beater, cDNA+bb: cDNA synthesised from RNA extracted with bead-beater, cDNA-bb: cDNA synthesised from RNA extracted without bead-beater.
(PDF)

Figure S3 **Rarefaction curves of the samples treated with ‘Initial Filtering’.** The curves show the expected number of species retrieved (*y*-axis) as a function of number of reads sampled (*x*-axis).
(PDF)

Figure S4 **Quality score profiles of the reads assigned to the different species.** The quality scores are obtained from the reads remaining after ‘Initial Filtering’ (IF). (A) Sample DNA-Hap454, (B) Sample DNA-PRYM454.
(PDF)

Table S1 **Comparison of proportional species distribution between the LSU clone libraries and the species distribution in the mock community in terms of cell number and biomass.**
(DOCX)

Table S2 **Sequence difference count and % similarity in the SSU V4 region between the 11 haptophyte species present in the mock community.**
(DOCX)

Table S3 **Match between sequences denoised by AmpliconNoise and the reference sequences of the strains in the mock community.**
(DOCX)

File S1 **Description of preliminary study with clone libraries and Sanger sequencing, targeting LSU rDNA/rRNA.**
(DOCX)
