# Supplementary material for: 454 Pyrosequencing to Describe Microbial Eukaryotic Community Composition, Diversity and Relative Abundance: A Test for Marine Haptophytes
Source: PLoS One. 2013 Sep 12;8(9):e74371. doi: 10.1371/journal.pone.0074371 (PMC3771978; doi:10.1371/journal.pone.0074371)
Supplement: Figure S1 — Agarose gel loaded with PCR products for clone libraries. From left to right: Ladder (O’GeneRuler 100 bp Plus, Fermentas), template cDNA-bb (from RNA extracted without bead-beater), cDNA+bb (from RNA extracted with bead-beater), DNA-bb (DNA extracted without bead-beater), DNA+bb (DNA extracted with bead-beater). (PDF) [file pone.0074371.s001.pdf]

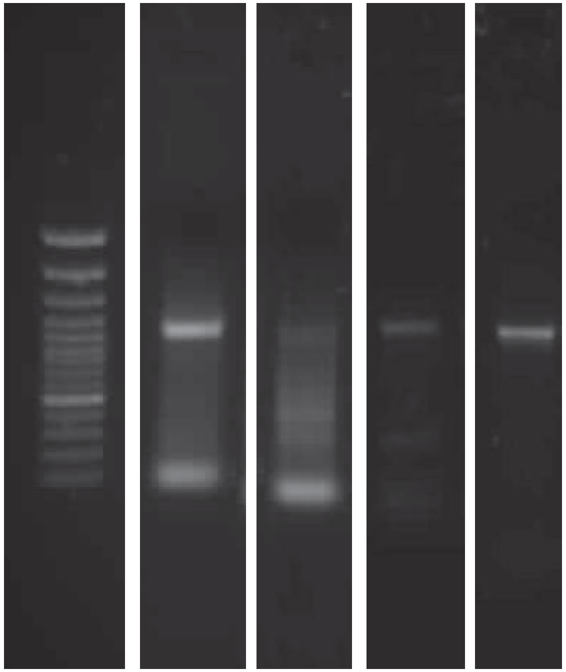

Figure S1. Agarose gel loaded with PCR products for clone libraries. From left to right: Ladder (O'GeneRuler 100bp plus, Fermentas), template cDNA-bb (from RNA extracted without bead-beater), cDNA+bb (from RNA extracted with bead-beater), DNA-bb (DNA extracted without bead-beater), DNA+bb (DNA extracted with bead-beater).
