# Supplementary material for: 454 Pyrosequencing to Describe Microbial Eukaryotic Community Composition, Diversity and Relative Abundance: A Test for Marine Haptophytes
Source: PLoS One. 2013 Sep 12;8(9):e74371. doi: 10.1371/journal.pone.0074371 (PMC3771978; doi:10.1371/journal.pone.0074371)
Supplement: Figure S2 — Proportional species abundance in LSU rDNA/rRNA D1–D2 clone libraries. Compared to the initial distribution of species in terms of cell number (first row) and biomass (second row) in the mock community. DNA+bb: DNA extracted with bead-beater, DNA-bb: DNA extracted without bead-beater, cDNA+bb: cDNA synthesised from RNA extracted with bead-beater, cDNA-bb: cDNA synthesised from RNA extracted without bead-beater. (PDF) [file pone.0074371.s002.pdf]

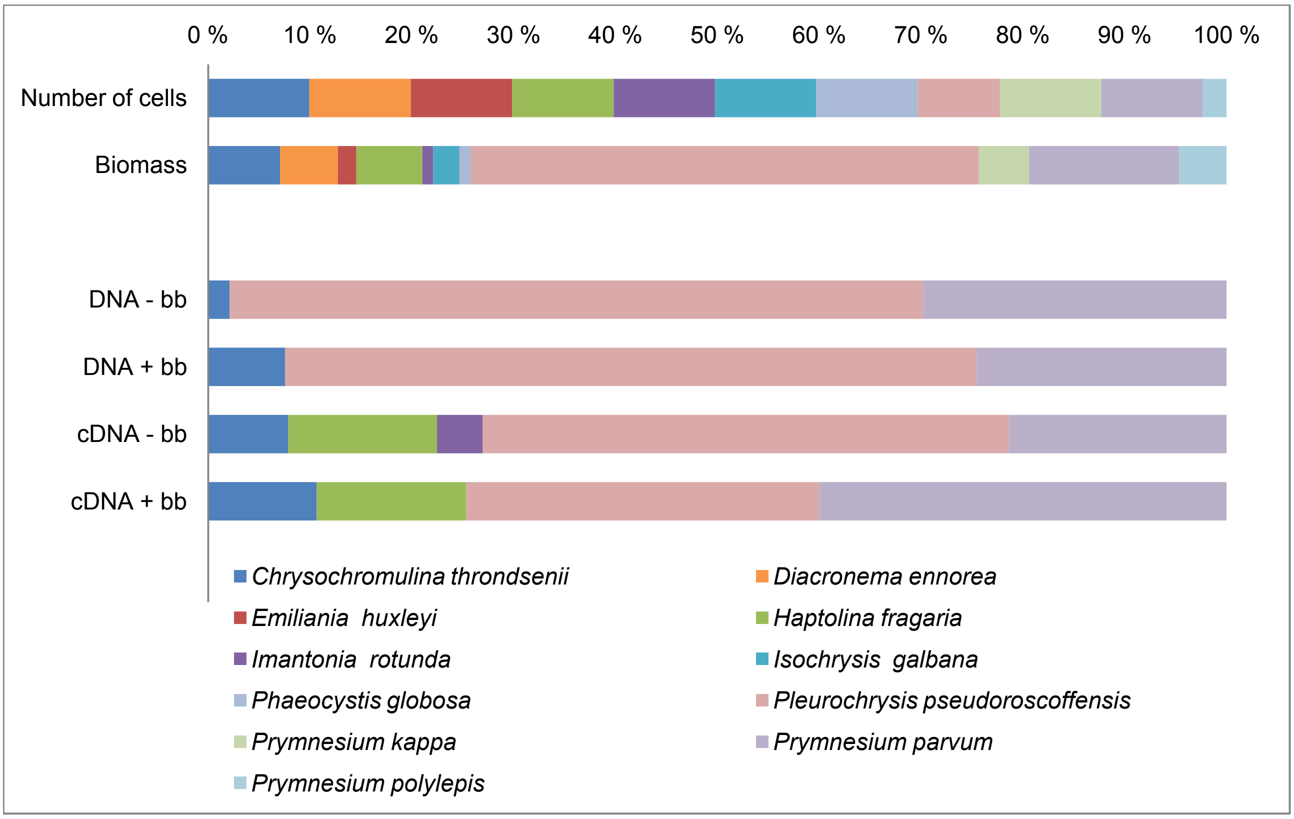

Figure S2. Proportional species abundance in LSU D1-D2 libraries. Compared to the initial distribution of species in terms of cell number (first row) and biomass (second row) in the mock community. DNA+bb: DNA extracted with bead-beater, DNA-bb: DNA extracted without bead-beater, cDNA+bb: cDNA synthesised from RNA extracted with bead-beater, cDNA-bb: cDNA synthesised from RNA extracted without bead-beater.
