# Supplementary material for: 454 Pyrosequencing to Describe Microbial Eukaryotic Community Composition, Diversity and Relative Abundance: A Test for Marine Haptophytes
Source: PLoS One. 2013 Sep 12;8(9):e74371. doi: 10.1371/journal.pone.0074371 (PMC3771978; doi:10.1371/journal.pone.0074371)
Supplement: Figure S3 — Rarefaction curves of the samples treated with ‘Initial Filtering’. The curves show the expected number of species retrieved (y-axis) as a function of number of reads sampled (x-axis). (PDF) [file pone.0074371.s003.pdf]

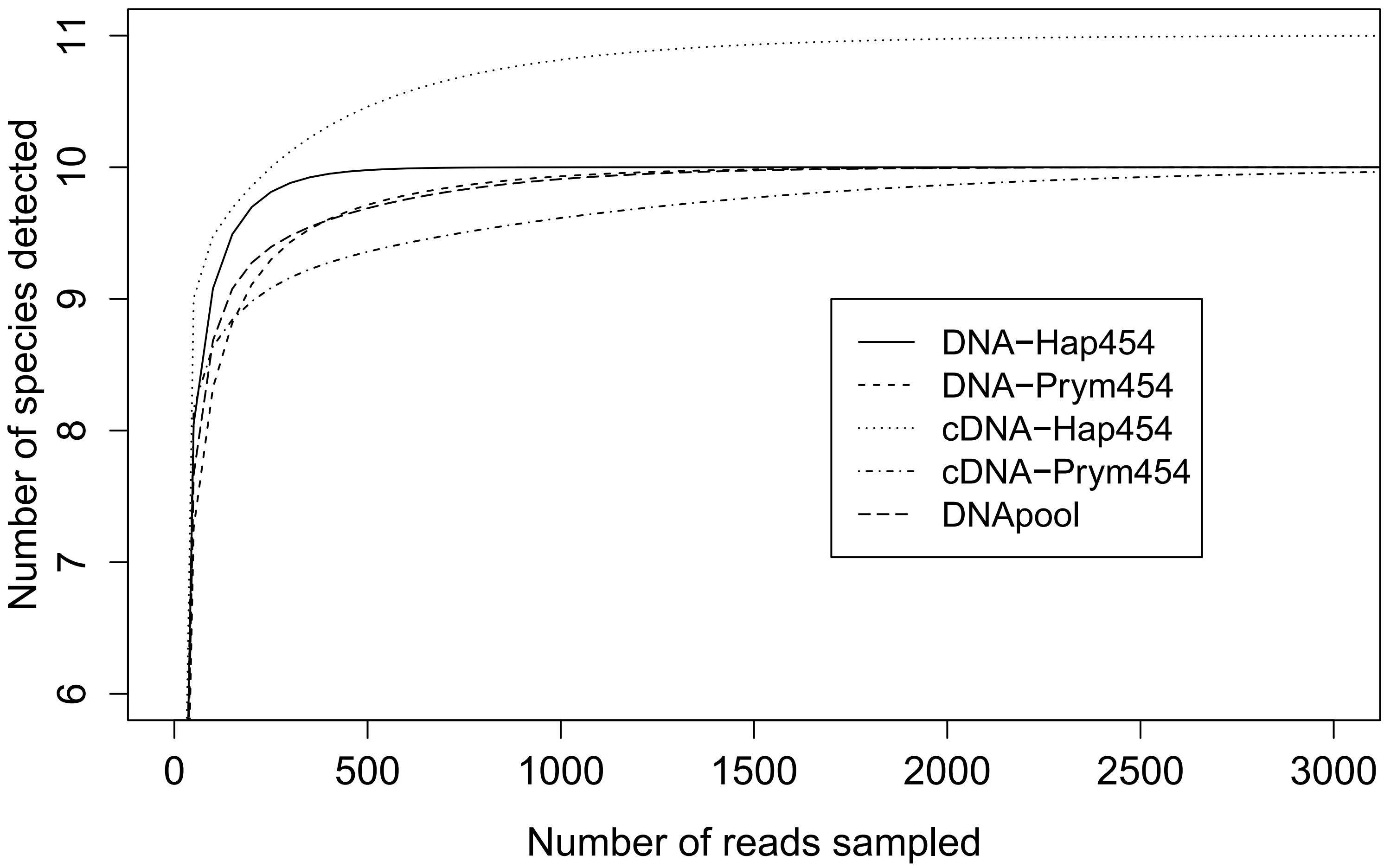

Figure S3. Rarefaction curves of the samples treated with Initial Filtering. The curves show the expected number of species retrieved (y-axis) as a function of number of reads sampled (x-axis).
