# Supplementary figures and images for: 454 Pyrosequencing to Describe Microbial Eukaryotic Community Composition, Diversity and Relative Abundance: A Test for Marine Haptophytes
Source: PLoS One. 2013 Sep 12;8(9):e74371. doi: 10.1371/journal.pone.0074371 (PMC3771978; doi:10.1371/journal.pone.0074371)

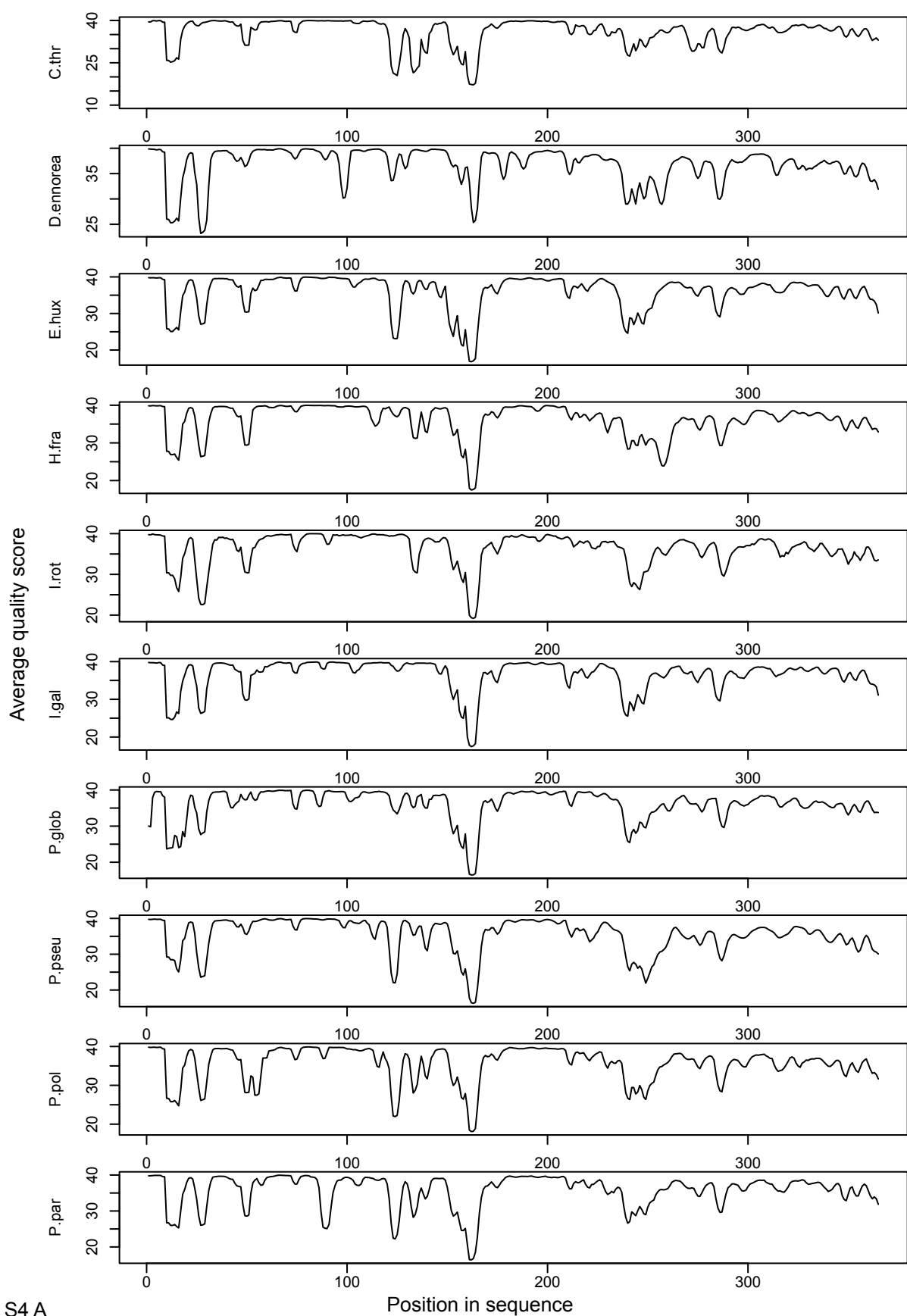

Figure S4 A

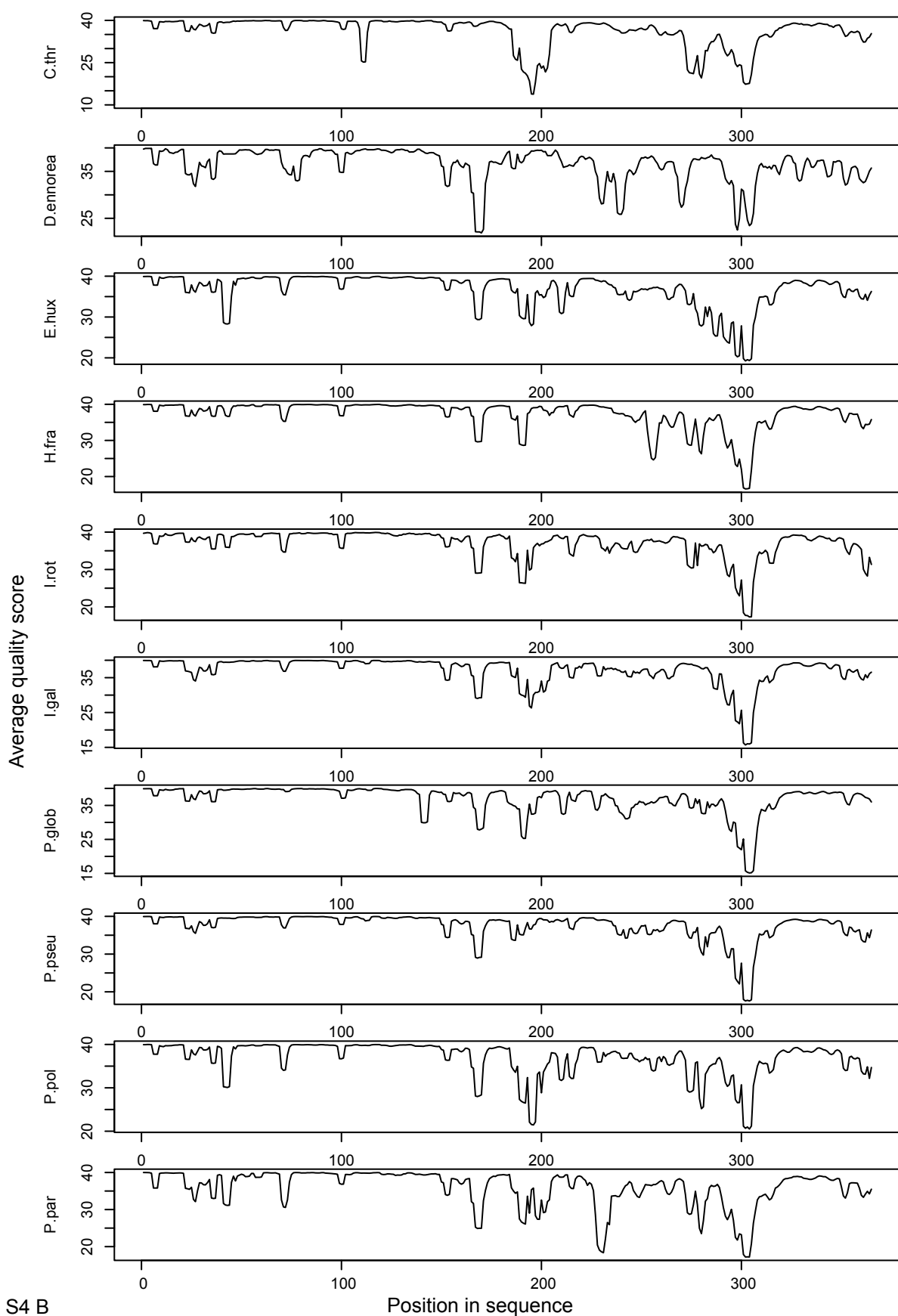

Figure S4 B

Supplement: Figure S4 — Quality score profiles of the reads assigned to the different species. The quality scores are obtained from the reads remaining after ‘Initial Filtering’ (IF). (A) Sample DNA-Hap454, (B) Sample DNA-PRYM454. (PDF) [file pone.0074371.s004.pdf]
