# Supplementary material for: 454 Pyrosequencing to Describe Microbial Eukaryotic Community Composition, Diversity and Relative Abundance: A Test for Marine Haptophytes
Source: PLoS One. 2013 Sep 12;8(9):e74371. doi: 10.1371/journal.pone.0074371 (PMC3771978; doi:10.1371/journal.pone.0074371)
Supplement: Table S1 — Comparison of proportional species distribution between the LSU clone libraries and the species distribution in the mock community in terms of cell number and biomass. (DOCX) [file pone.0074371.s005.docx]

Table S1. Comparison of proportional species distribution between the LSU clone libraries and the species distribution in the mock community in terms of cell number and biomass.

| Compared to:   Sample: | Initial distribution of biomass from the different species | Initial distribution by cell number | DNA + bb | cDNA – bb | cDNA + bb |
| --- | --- | --- | --- | --- | --- |
| DNA - bb (47) | p = **0.01** | p = **7e-13** | p = 0.53 | p = **0.005** |  |
| DNA + bb (53) | p = **0.02** | p = **3e-13** |  |  | p = **0.0003** |
| cDNA - bb (89) | p = **0.01** | p = **3e-15** |  |  | p = **0.02** |
| cDNA + bb (75) | p = **0.0002** | p = **3e-13** |  |  |  |

p-values from comparison of the species distributions in the clone libraries by the Fisher exact test. Significant (< 0.05) p-values values in bold type. Number of clones in each library are given in parentheses.
