# Supplementary material for: 454 Pyrosequencing to Describe Microbial Eukaryotic Community Composition, Diversity and Relative Abundance: A Test for Marine Haptophytes
Source: PLoS One. 2013 Sep 12;8(9):e74371. doi: 10.1371/journal.pone.0074371 (PMC3771978; doi:10.1371/journal.pone.0074371)
Supplement: Table S2 — Sequence difference count and % similarity in the SSU V4 region between the 11 haptophyte species present in the mock community. (DOCX) [file pone.0074371.s006.docx]

Table S2 (A). Sequence difference count (upper triangular), and % similarity (lower triangular) in the SSU rDNA region spanned by primer pair Hap454, for the 11 haptophyte species present in the mock community.

|  | ***Emiliania huxleyi*** | ***Isochrysis galbana*** | ***Pleurochrysis pseudoroscoffensis*** | ***Imantonia rotunda*** | ***Prymnesium kappa*** | ***Prymnesium parvum*** | ***Prymnesium polylepis*** | ***Haptolina fragaria*** | ***Chrysochromulina throndsenii*** | ***Phaeocystis globosa*** | ***Diacronema ennorea*** |
| --- | --- | --- | --- | --- | --- | --- | --- | --- | --- | --- | --- |
| ***Emiliania huxleyi*** | ID | 18 | 23 | 30 | 17 | 20 | 18 | 17 | 14 | 27 | 70 |
| ***Isochrysis galbana*** | 95.0 % | ID | 32 | 31 | 22 | 25 | 24 | 23 | 24 | 34 | 77 |
| ***Pleurochrysis pseudoroscoffensis*** | 93.6 % | 91.1 % | ID | 25 | 17 | 21 | 19 | 22 | 24 | 37 | 70 |
| ***Imantonia rotunda*** | 91.4 % | 91.4 % | 92.8 % | ID | 20 | 23 | 20 | 24 | 27 | 43 | 76 |
| ***Prymnesium kappa*** | 95.3 % | 93.9 % | 95.3 % | 94.5 % | ID | 5 | 6 | 7 | 12 | 33 | 72 |
| ***Prymnesium parvum*** | 94.4 % | 93.1 % | 94.2 % | 93.6 % | 98.6 % | ID | 7 | 10 | 15 | 36 | 68 |
| ***Prymnesium polylepis*** | 95.0 % | 93.3 % | 94.7 % | 94.5 % | 98.3 % | 98.0 % | ID | 7 | 15 | 34 | 69 |
| ***Haptolina fragaria*** | 95.3 % | 93.6 % | 93.9 % | 93.4 % | 98.0 % | 97.2 % | 98.0 % | ID | 13 | 33 | 70 |
| ***Chrysochromulina throndsenii*** | 96.1 % | 93.3 % | 93.3 % | 92.5 % | 96.6 % | 95.8 % | 95.8 % | 96.4 % | ID | 29 | 69 |
| ***Phaeocystis globosa*** | 92.5 % | 90.6 % | 89.8 % | 87.9 % | 90.9 % | 90.1 % | 90.6 % | 90.9 % | 92.0 % | ID | 71 |
| ***Diacronema ennorea*** | 79.7 % | 77.8 % | 80.0 % | 77.8 % | 79.4 % | 80.2 % | 80.0 % | 79.7 % | 80.0 % | 79.5 % | ID |

Table S2 (B). Sequence difference count (upper triangular) and % similarity (lower triangular) in the SSU rDNA region spanned by primer pair Prym454, for the 11 haptophyte species present in the mock community.

|  | ***Emiliania huxleyi*** | ***Isochrysis galbana*** | ***Pleurochrysis pseudoroscoffensis*** | ***Imantonia rotunda*** | ***Prymnesium kappa*** | ***Prymnesium parvum*** | ***Prymnesium polylepis*** | ***Haptolina fragaria*** | ***Chrysochromulina throndsenii*** | ***Phaeocystis globosa*** | ***Diacronema ennorea*** |
| --- | --- | --- | --- | --- | --- | --- | --- | --- | --- | --- | --- |
| ***Emiliania huxleyi*** | ID | 19 | 25 | 31 | 17 | 17 | 17 | 17 | 15 | 26 | 69 |
| ***Isochrysis galbana*** | 94.8 % | ID | 35 | 35 | 23 | 23 | 24 | 24 | 26 | 34 | 72 |
| ***Pleurochrysis pseudoroscoffensis*** | 93.2 % | 90.6 % | ID | 24 | 17 | 20 | 20 | 24 | 26 | 38 | 71 |
| ***Imantonia rotunda*** | 91.4 % | 90.6 % | 93.3 % | ID | 23 | 23 | 22 | 25 | 29 | 41 | 78 |
| ***Prymnesium kappa*** | 95.4 % | 93.8 % | 95.4 % | 93.8 % | ID | 4 | 7 | 9 | 14 | 32 | 73 |
| ***Prymnesium parvum*** | 95.4 % | 93.8 % | 94.6 % | 93.8 % | 98.9 % | ID | 5 | 9 | 15 | 32 | 70 |
| ***Prymnesium polylepis*** | 95.4 % | 93.5 % | 94.6 % | 94.1 % | 98.1 % | 98.6 % | ID | 8 | 17 | 32 | 69 |
| ***Haptolina fragaria*** | 95.4 % | 93.5 % | 93.5 % | 93.3 % | 97.5 % | 97.5 % | 97.8 % | ID | 14 | 30 | 71 |
| ***Chrysochromulina throndsenii*** | 95.9 % | 93.0 % | 93.0 % | 92.2 % | 96.2 % | 95.9 % | 95.4 % | 96.2 % | ID | 25 | 72 |
| ***Phaeocystis globosa*** | 93.0 % | 90.9 % | 89.8 % | 88.8 % | 91.4 % | 91.4 % | 91.4 % | 91.9 % | 93.3 % | ID | 73 |
| ***Diacronema ennorea*** | 81.3 % | 80.0 % | 81.3 % | 78.7 % | 80.8 % | 81.0 % | 81.3 % | 80.8 % | 81.3 % | 80.8 % | ID |
