# Supplementary material for: 454 Pyrosequencing to Describe Microbial Eukaryotic Community Composition, Diversity and Relative Abundance: A Test for Marine Haptophytes
Source: PLoS One. 2013 Sep 12;8(9):e74371. doi: 10.1371/journal.pone.0074371 (PMC3771978; doi:10.1371/journal.pone.0074371)
Supplement: Table S3 — Match between sequences denoised by AmpliconNoise and the reference sequences of the strains in the mock community. (DOCX) [file pone.0074371.s007.docx]

**Table S3.** Match between sequences denoised by AmpliconNoise and the reference sequences of the strains in the mock community.

| Species | DNA | | | | | | cDNA | | | | | | DNApool | | |
| --- | --- | --- | --- | --- | --- | --- | --- | --- | --- | --- | --- | --- | --- | --- | --- |
|  | Hap454 | | | Prym454 | | | Hap454 | | | Prym454 | | | Prym454 | | |
|  | Unique seq | Indels | Mis-matches | Unique seq | Indels | Mis-matches | Unique seq | Indels | Mis-matches | Unique seq | Indels | Mis-matches | Unique seq | Indels | Mismatches |
| *Chrysochromulina*  *_throndsenii* | 1 | 3 | 0 | 1 | 2 | 0 | 4 | 3 | 1 | 2 | 6 | 6 | 1 | 4 | 0 |
| *Diacronema ennorea* | 1 | 1 | 0 | 1 | 0 | 0 | 2 | 0 | 0 | 1 | 3 | 5 | 1 | 0 | 0 |
| *Emiliania huxleyi* | 1 | 2 | 0 | 1 | 1 | 0 | 5 | 1 | 0 | 3 | 1 | 0 | 1 | 0 | 0 |
| *Haptolina fragaria* | 1 | 0 | 0 | 1 | 1 | 0 | 2 | 0 | 0 | 1 | 1 | 0 | 1 | 1 | 0 |
| *Imantonia rotunda* | 1 | 2 | 0 | 1 | 2 | 0 | 1 | 2 | 0 | 1 | 2 | 0 | 1 | 2 | 0 |
| *Isochrysis galbana* | 1 | 4 | 1 | 1 | 3 | 1 | 4 | 2 | 1 | 1 | 2 | 1 | 3 | 3 | 1 |
| *Phaeocystis globosa* | 1 | 0 | 0 | 1 | 0 | 0 | 5 | 0 | 0 | 1 | 0 | 0 | 2 | 0 | 0 |
| *Pleurochrysis pseudoroscoffensis* | 1 | 0 | 0 | 1 | 1 | 0 | 2 | 0 | 0 | 1 | 1 | 0 | 1 | 1 | 0 |
| *Prymnesium kappa* | NA | NA | NA | NA | NA | NA | NA | NA | NA | NA | NA | NA | NA | NA | NA |
| *Prymnesium parvum* | 1 | 1 | 0 | 1 | 1 | 0 | 4 | 2 | 0 | 1 | 1 | 0 | 1 | 1 | 0 |
| *Prymnesium polylepis* | 1 | 1 | 0 | 1 | 1 | 0 | 2 | 0 | 0 | 1 | 1 | 0 | 1 | 1 | 0 |

Unique: Number of unique sequences assigned to the species. Indels: Number of insertions or deletions in the AmpliconNoise-treated sequence compared to the reference sequence. Mismatches: Number of mismatches between AmpliconNoise-treated sequence and reference sequence. Where more than one unique sequence was assigned to a species, ‘indels’ and ‘mismatches’ are given for the most abundant sequence.
