# Supplementary material for: 454 Pyrosequencing to Describe Microbial Eukaryotic Community Composition, Diversity and Relative Abundance: A Test for Marine Haptophytes
Source: PLoS One. 2013 Sep 12;8(9):e74371. doi: 10.1371/journal.pone.0074371 (PMC3771978; doi:10.1371/journal.pone.0074371)
Supplement: File S1 — Description of preliminary study with clone libraries and Sanger sequencing, targeting LSU rDNA/rRNA. (DOCX) [file pone.0074371.s008.docx]

# Supporting Information File S1

# Preliminary study with partial LSU rDNA/rRNA clone libraries and Sanger sequencing

## Mock community of haptophytes

A mock community consisting of 11 strains of different haptophyte species were produced as described in the main paper Egge et al.

## DNA and RNA extraction

The algal pellet was thawed on ice, and the cells were resuspended until homogenized. DNA and RNA were extracted with the RNA NucleoSpin II kit (Macherey-Nagel, Düren, Germany), with the NucleoSpin RNA/DNA Buffer set and addition of β-mercaptoethanol in the lysing step. Two extraction methods were tested for the clone libraries; one where the cells were mixed with lysis buffer without mechanical disruption in a bead-beater, and one including mechanical disruption in a bead-beater during the lysis step. Without bead-beating: 350 µl of lysis buffer and 3.5 µl β-mercaptoethanol was added to each of five parallel eppendorf tubes containing 350 µl of the sample, vortexed for 30 s, and RNA and DNA was extracted according to the protocol from the manufacturer. With bead-beating: 700 µl of the sample was mixed with 700 µl lysis buffer and 70 µl β-mercaptoethanol, and 735 µl of this mixture were added to each of two Lysing Matrix D tubes containing 1.4 mm ceramic beads (MP Biomedicals, Solon, Ohio). The samples were homogenized in a bead-beater (Precellys 24, Bertin technologies, Paris) at 6500 rpm for 2 x 20 s with a 15 s break. The lysate was distributed into two extraction columns, and the RNA and DNA extraction performed according to the protocol from the manufacturer.

## LSU ribosomal DNA and RNA/cDNA clone library construction

In the following, the terms DNA-bb, DNA+bb, cDNA-bb and cDNA+bb will be used to refer to the templates of DNA extracted without bead-beater (DNA-bb), with bead-beater (DNA+bb), and of cDNA transcribed from RNA extracted without (cDNA-bb) and with bead-beater (cDNA+bb).

LSU rDNA fragments starting in the ITS2, and including the D1-D2 domain were PCR amplified from the four samples described above using the forward haptophyte-specific primer *Hapto_4* (ATGGCGAATGAAGCGGGC) [1] and the reverse general eukaryote primer *Euk_34r* (GCATCGCCAGTTCTGCTTACC) [1]. PCR reactions (30 cycles of 94° C for 45 s, 51° C for 45 s, 68° C for 60 s, and initial denaturation and final extension step) were run using the proofreading Taq DNA polymerase (5prime, Hamburg, Germany). Each reaction contained 5 µl PCR buffer, 5 µl enchancer (5prime), 0.2 mM dNTP, 0.2 µM of each primer, c. 10 ng template, and nuclease-free H_2_O to a total volume of 50 µl. For each of the clone libraries, PCR was run in six separate reactions that were pooled before the clone libraries were constructed. The length of the PCR products was checked by agarose gel electrophoresis. The products were run on a 1% agarose gel (SeaKem, Philadelphia, PA, USA), cut out of the gel and purified using the Wizard SV gel and PCR-clean up system kit (Promega, Madison, WI, USA), according to the protocol from the manufacturer.

Four clone libraries were constructed using the TOPO TA Cloning kit with pCR4-TOPO vectors and competent *E. coli* cells (Invitrogen), according to the manufacturer’s protocol. Fifty-five colonies from each of clone libraries DNA-bb and DNA+bb were subjected to direct PCR with M13F/R primers and Dynazyme II polymerase in the following PCR cycle: 94 °C for 10 min, then 25 cycles of 1 min at 94 °C, 1 min 55 °C, 1 min 72 °C, and a final extension step at 72 °C for 10 mins. The PCR products were sequenced in the forward direction with Hapto_4 as primer, using Applied Biosystems BigDye Terminator and an Applied Biosystems 3730 analyser (Applied Biosystems, Foster City, CA, USA) at the Department of Biology, University of Oslo, Norway. For clone libraries cDNA-bb and cDNA+bb, 96 colonies were picked and incubated on agar in 96-well microtiter plates prior to plasmid preparation and sequencing in the forward direction with primer M13F at LGC Genomics, Berlin, Germany. All sequences were inspected visually and edited using BioEdit. Each sequence was assigned to one of the 11 species in the mock community by BLAST [2] against the NCBI database. The accession numbers of the partial LSU sequences of the strains used in this study are listed in Table 1 in the main paper.

To test whether the proportions of the different species in the clone libraries were significantly different from each other, and from the initial proportion of species in terms of biomass and cell number in the culture mix, we used the Fisher exact test [3].

## Results

The PCR products from cDNA produced from the RNA extracted with a bead-beater step showed weaker bands and more smear on an agarose gel compared to cDNA produced from RNA extracted without bead-beater (Figure S1). This suggests that the bead-beating step caused fragmentation or degradation of the RNA. Bead-beating did not appear to affect the DNA as template.

From each clone library we obtained between 47-89 partial LSU rDNA/rRNA sequences (Table S1). The proportional species distribution in each clone library is shown in Figure S2. In the culture mix, all species were represented with about equal cell numbers (first line in Figure S2, except *Prymnesium polylepis*), but varying biomass (second line) as estimated from cell volume (Table 1 in main paper). Homogenization with a bead-beater during nucleic acid extraction did not increase the number of species retrieved (3 or 4 species with, and 3 or 5 without bead-beating), and did not significantly affect the proportional species distribution observed in the clone libraries (Table S1; DNA-bb vs. DNA+bb and cDNA-bb vs. cDNA+bb, Fisher’s exact test, both p-values > 0.2). The 454-pyrosequencing was therefore produced from the samples extracted without a bead-beating step.

The species distributions in all clone libraries were significantly different from the initial cell number or biomass distributions (Table S1; Fisher’s exact test, all p-values <0.0001). The species represented by highest biomasses in the mock community, (*Pleurochrysis pseudoroscoffensis, Prymnesium parvum, Chrysochromulina throndsenii* and *Haptolina fragaria*), were also represented in the same rank order in the clone libraries. However, the clone libraries could only recover 3-5 out of the 11 species initially present in the mock community when analysing 47-89 sequences per sample (in total 264 clones). Sequences from *Prymnesium polylepis, Emiliania huxleyi, Phaeocystis globosa, Isochrysis galbana,* and *Diacronema ennorea* were not retrieved in any of the clone libraries, despite 100% match of the primers to available haptophyte ITS2-LSU rDNA sequences.

The species distributions were significantly different in the DNA-based clone libraries compared to the cDNA-based (Table S1; Fishers exact test, p-value < 0.05). More species were recorded in the cDNA (4 or 5) than in the DNA (3) clone libraries. *H. fragaria* appeared in both cDNA clone libraries, but not in the DNA clone libraries, and *Imantonia rotunda* appeared only in the cDNA clone library constructed from RNA extracted without bead-beater.

Table S1. Comparison of proportional species distribution between the LSU clone libraries and the species distribution in the mock community in terms of cell number and biomass.

| Compared to:   Sample: | Initial distribution of biomass from the different species | Initial distribution by cell number | DNA + bb | cDNA – bb | cDNA + bb |
| --- | --- | --- | --- | --- | --- |
| DNA - bb (47) | p = **0.01** | p = **7e-13** | p = 0.53 | p = **0.005** |  |
| DNA + bb (53) | p = **0.02** | p = **3e-13** |  |  | p = **0.0003** |
| cDNA - bb (89) | p = **0.01** | p = **3e-15** |  |  | p = **0.02** |
| cDNA + bb (75) | p = **0.0002** | p = **3e-13** |  |  |  |

p-values from comparison of the species distributions in the clone libraries by the Fisher exact test. Significant (< 0.05) p-values values in bold type. Number of clones in each library are given in parentheses.

**Figure S1. Agarose gel loaded with PCR products for clone libraries.** From left to right: Template cDNA-bb (from RNA extracted without bead-beater), cDNA+bb (from RNA extracted with bead-beater), DNA-bb (DNA extracted without bead-beater), DNA+bb (DNA extracted with bead-beater).

**Figure S2. Proportional species abundance in LSU D1-D2 libraries**. Compared to the initial distribution of species in terms of cell number (first row) and biomass (second row) in the mock community. DNA+bb: DNA extracted with bead-beater, DNA-bb: DNA extracted without bead-beater, cDNA+bb: cDNA synthesised from RNA extracted with bead-beater, cDNA-bb: cDNA synthesised from RNA extracted without bead-beater.

## References

1. Liu H, Probert I, Uitz J, Claustre H, Aris-Brosou S, et al. (2009) Extreme diversity in noncalcifying haptophytes explains a major pigment paradox in open oceans. Proc Natl Acad Sci USA 106: 12803–12808.

2. Altschul SF, Madden TL, Schäffer AA, Zhang J, Zhang Z, et al. (1997) Gapped BLAST and PSI-BLAST: a new generation of protein database search programs. Nucleic Acids Res 25: 3389–3402.

3. Agresti A (1992) A Survey -of Exact Inference for Contingency Tables. Statistical Science 7: 131–153.
